# Integration of immunoinformatics and cheminformatics to design and evaluate a multitope vaccine against *Klebsiella pneumoniae* and *Pseudomonas aeruginosa* coinfection

**DOI:** 10.3389/fmolb.2023.1123411

**Published:** 2023-02-14

**Authors:** Ahmed M. Gouda, Mohamed A. Soltan, Khalid Abd-Elghany, Ashraf E. Sileem, Hanan M. Elnahas, Marwa Abdel-Monem Ateya, Mahmoud H. Elbatreek, Khaled M. Darwish, Hanin A. Bogari, Manar O. Lashkar, Mohammed M. Aldurdunji, Sameh S. Elhady, Tarek A. Ahmad, Ahmed Mohamed Said

**Affiliations:** ^1^ Department of Pharmacy Practice, Faculty of Pharmacy, Zagazig University, Zagazig, Egypt; ^2^ Department of Microbiology and Immunology, Faculty of Pharmacy, Sinai University-Kantara Branch, Ismailia, Egypt; ^3^ Department of Microbiology-Microbial Biotechnology, Egyptian Drug Authority, Giza, Egypt; ^4^ Department of Chest Diseases, Faculty of Medicine, Zagazig University, Zagazig, Egypt; ^5^ Department of Pharmaceutical and Industrial Pharmacy, Faculty of Pharmacy, Zagazig University, Zagazig, Egypt; ^6^ Department of Clinical Pathology, Faculty of Medicine, Zagazig University, Zagazig, Egypt; ^7^ Department of Pharmacology and Toxicology, Faculty of Pharmacy, Zagazig University, Zagazig, Egypt; ^8^ Department of Medicinal Chemistry, Faculty of Pharmacy, Suez Canal University, Ismailia, Egypt; ^9^ Department of Pharmacy Practice, Faculty of Pharmacy, King Abdulaziz University, Jeddah, Saudi Arabia; ^10^ Department of Clinical Pharmacy, College of Pharmacy, Umm Al-Qura University, Makkah, Saudi Arabia; ^11^ Department of Natural Products, Faculty of Pharmacy, King Abdulaziz University, Jeddah, Saudi Arabia; ^12^ Center for Artificial Intelligence in Precision Medicines, King Abdulaziz University, Jeddah, Saudi Arabia; ^13^ Library Sector, Bibliotheca Alexandrina, Alexandria, Egypt

**Keywords:** *Klebsiella pneumoniae*, *Pseudomonas aeruginosa*, immunoinformatics, multitope vaccine, industries development, drug discovery, public health

## Abstract

**Introduction:** *Klebsiella pneumoniae* (*K. pneumoniae*) and *Pseudomonas aeruginosa* (*P. aeruginosa*) are the most common Gram-negative bacteria associated with pneumonia and coinfecting the same patient. Despite their high virulence, there is no effective vaccine against them.

**Methods:** In the current study, the screening of several proteins from both pathogens highlighted FepA and OmpK35 for *K. pneumonia* in addition to HasR and OprF from *P. aeruginosa* as promising candidates for epitope mapping. Those four proteins were linked to form a multitope vaccine, that was formulated with a suitable adjuvant, and PADRE peptides to finalize the multitope vaccine construct. The final vaccine’s physicochemical features, antigenicity, toxicity, allergenicity, and solubility were evaluated for use in humans.

**Results:** The output of the computational analysis revealed that the designed multitope construct has passed these assessments with satisfactory scores where, as the last stage, we performed a molecular docking study between the potential vaccine construct and *K. pneumonia* associated immune receptors, TLR4 and TLR2, showing affinitive to both targets with preferentiality for the TLR4 receptor protein. Validation of the docking studies has proceeded through molecular dynamics simulation, which estimated a strong binding and supported the nomination of the designed vaccine as a putative solution for *K. pneumoniae* and *P. aeruginosa* coinfection. Here, we describe the approach for the design and assessment of our potential vaccine.

## Introduction

*Klebsiella pneumoniae* is a Gram-negative bacteria that cause hospital and society-induced infections all over the world (Liao et al., 2020). It is responsible for 0.5%–5.0% of all cases of pneumonia, which usually leads to a high incidence of complications and an increased rate of mortality that reaches more than half of the patients (Okada et al., 2010). The control of *K. pneumoniae* infections is a complicated task due to the pathogen’s increasing multi-drug resistance capacity, the escape of infection control strategies, the emergence of hypervirulent strains, the oxidative stress induced by *Klebsiella* to suppress the immune system, and the tendency of infection recurrence. These together urged the need to prevent pathogens by immunoprophylactic means rather than to treat it (Ahmad et al., 2012). *Pseudomonas aeruginosa* is regularly classified as an opportunistic bacteria and several reports explain that it is considered the most common bacterium associated with nosocomial infections and ventilator-associated pneumonia (Barbier et al., 2013). Coinfection is a condition that can be defined as the concurrent infection of the host caused by several pathogens. An obvious case for such connectivity is that of *K. pneumoniae* and *P. aeruginosa* which have been frequently isolated from the same patient (Jones-Nelson et al., 2018). It was reported that in 79.4% of patients suffering acute *K. pneumoniae* pneumonia infection, one or more additional bacteria, predominantly *P. aeruginosa* has been detected (Okada et al., 2009). Simultaneously, the formation of biofilms of *K. pneumoniae* and *P. aeruginosa* coinfection enhances the persistence of infection of wounds, leading to chronic ulcers. This is mostly due to the biofilm’s resistance to the host immunity and to the use of antimicrobials (Childers et al., 2013).

It is worth mentioning that continuous misuse of antibiotics in some countries has remarkably contributed to the emergence of several resistant strains, that are easily disseminated all over the world by travelers (Ben et al., 2019). In the last 2 years, a pan-alarm to humanity arose that nobody is far away from extensively drug-resistant (XDR) strains (Wright, 2007). On the other hand, *P. aeruginosa* was also reported for its new mechanism of biofilm-mediated resistance, the generation of multidrug-tolerant persister cells, as well as, its responsibility for infection relapse (Pang et al., 2019). The case of coinfection of pathogens complicated the trials for treatment (Rahmat Ullah et al., 2021), highlighting the urgent necessity to prevent such pathogens by immunoprophylactic strategies. Despite several trials being adopted over the last 50 years, there is no vaccine in the market against *K. pneumoniae* (Ahmad et al., 2012; Assoni et al., 2021) nor *P. aeruginosa* (Cabral et al., 2020) up till now.

In the early stages of vaccine production against *Klebsiella* and *Pseudomonas*, researchers tended to develop killed or digested whole-cell vaccines of the pathogens (Ahmad et al., 2012; Assoni et al., 2021). However, due to the immune noise induced by mixes of bacterial epitopes, the development of vaccines oriented research towards subunit vaccines, either based on bacterial cell wall polysaccharides or different cellular proteins. Upon the advancement in molecular techniques, the protein subunits were preferred and their way was followed to produce the third and fourth-generation vaccines trend (Ahmad et al., 2012). The major proteins that were used to produce vaccines are the outer membrane proteins and adhesins. Since outer membrane proteins in Gram-negative bacteria are the outermost bacterial facade that comes in contact with the immune cells (Baliga et al., 2018). Several structural outer membrane proteins were applied for vaccine production, such as the OmpA.

Inorganic iron has important roles in DNA synthesis and cellular respiration in bacterial cells (Hood and Skaar, 2012). Throughout the primary stages of bacterial infection, the host, as a defensive mechanism, uses different mechanisms to sequester and starve the bacterial cells for iron, in an approach called nutritional immunity. On the other hand, Gram-negative pathogens have developed an opposing strategy which is the membrane siderophore proteins that can capture low amounts of iron or even organic iron from the host’s heme that allows for bacterial survival despite the nutritional immunity state (Richard et al., 2019). That is why these siderophores are highly proposed to be building blocks for vaccines against Gram-negative bacteria (Sainz-Mejías et al., 2020; Assoni et al., 2021).

The development of reverse vaccinology and immunoinformatics approaches alongside other computational tools for *in silico* assessment has fastened the process of vaccine development in an economical way (Soltan et al., 2020). In the current study, several probable vaccine blocks were screened against the relevant pathogens in order to propose the predicted most potent epitopes. Those single epitopes were integrated into a final multitope construct and assessed for their physicochemical and immunological properties. Finally, the potential vaccine construct was evaluated for its docking ability with human immune receptors through molecular docking-coupled dynamics simulations.

## Materials and methods

**FIGURE 1:**
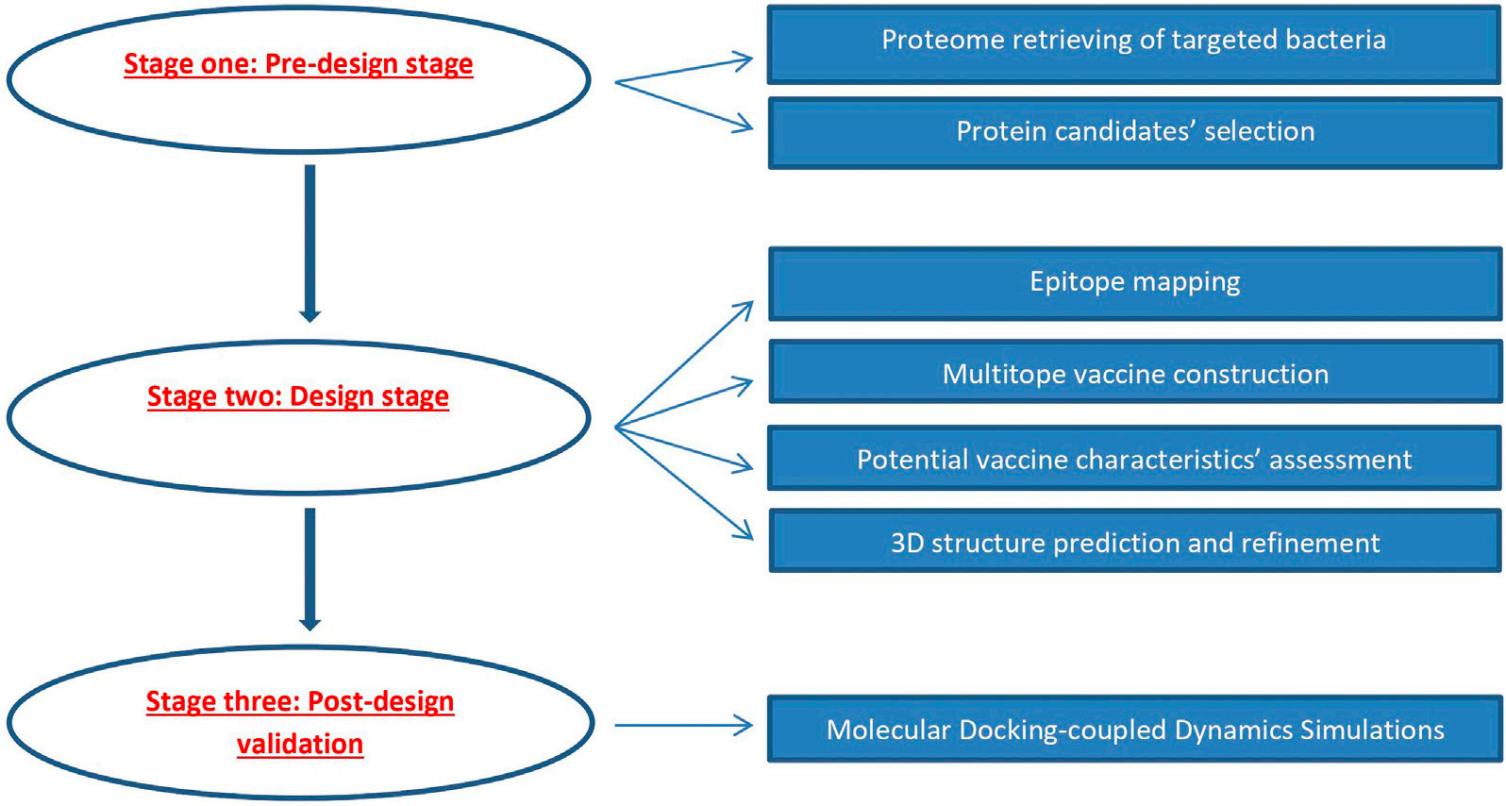
Graphical representation of the applied strategy for designing a chimeric epitope vaccine against *Klebsiella pneumoniae* and *Pseudomonas aeruginosa* coinfection.

An overview of the applied strategy for a potential multitope vaccine design against *K. pneumoniae* and *P. aeruginosa* coinfection is shown in Figure 1.

### Data retrieval and vaccine candidates’ selection

The reference proteomes of *K. pneumoniae* (strain ATCC 700721/MGH 78578) and *P. aeruginosa* (strain ATCC 15692/DSM 22644/CIP 104116/JCM 14847/LMG 12228/1C/PRS 101/PAO1) were downloaded from the UniProt webserver (https://www.uniprot.org/) under the proteome ID of UP000000265 and UP000002438, respectively. As mentioned in the introduction section, the current study aims to design an epitope-based vaccine through the filtration of protein candidates belonging to the outer membrane and iron uptake proteins. Therefore, we selected nine *K. pneumoniae* protein candidates namely FepA, FepB, FepC, FhuA, FhuF, FuR (iron uptake proteins), OmpA, OmpC, and OmpF (outer membrane proteins), and filtered them through their antigenicity score estimated by VaxiJen v2.0 (Doytchinova and Flower, 2007) with the cutoff score of 0.4 (the threshold value of bacterial antigenic proteins). The assessment of the antigenicity score revealed that there were 8 antigenic proteins, out of the selected 9 ones therefore we selected the top 2 proteins (one protein from each category) based on their antigenicity score where the final 2 protein candidates of *K. pneumoniae* were FepA and OmpF with antigenicity scores of 0.76 and 0.81 respectively. Moving to *P. aeruginosa*, we followed the same approach where six protein candidates namely FoxA, FpvA, HasR, HitA (iron uptake proteins), OprF, and OprH (outer membrane proteins) were filtered and 2 proteins (also one from each category) namely HasR and OprF with the antigenicity scores of 0.59 and 0.8 respectively were selected as our final candidates for *P. aeruginosa*.

### T and B cell epitopes mapping

The filtered 4 proteins from the previous step were uploaded to SignalP- 5.0 server (Almagro Armenteros et al., 2019) to predict the location of signal peptides. Following that, the mature polypeptides were analyzed for their T and B cell epitopes through the Immune Epitope Database (IEDB) (Dhanda et al., 2019). Firstly, we mapped CTLs for the protein candidates using the HLA allele reference set, which provided more than 97% in terms of population coverage (Weiskopf et al., 2013), and the NetMHCpan EL 4.1 prediction tool (that was recommended by the IEDB database). Secondly, we mapped for HTLs against the HLA reference set to cover more than 99% in terms of population coverage (Greenbaum et al., 2011) and used IEDB recommended 2.22 as a prediction method. Furthermore, HTL peptides were assessed for their ability to induce several cytokines such as IFN-gamma (Dhanda et al., 2013b), IL-4 (Dhanda et al., 2013a), IL-10 (Nagpal et al., 2017), IL-6, and IL-13 (Jain et al., 2022). The last analysis for HTLs and CTLs was the conservancy prediction where multiple sequence alignment against the corresponding proteins in other reference sequences was employed to validate the conservancy of the selected epitopes. The last set of epitopes; namely BCLs were finally estimated through IEBD using the BepiPred-2.0 prediction method (Jespersen et al., 2017). Following prediction, the estimated epitopes were filtered based on the consideration of several characteristics such as the number of reacting alleles (to achieve a high population coverage percentage), conservancy percentage, and antigenicity score.

### Multitope vaccine construction

Following the detection of B and T cell epitopes, we selected top-ranking single epitopes to initiate a multitope vaccine construct representing the epitopes of 4 proteins of our 2 bacteria of interest. Therefore, the best 2 candidates of CTL, HTL, and BCL epitopes per each protein were linked through GGGS, GPGPG, and KK amino acid linkers, respectively, in order to apply *in vivo* separation of the joined epitopes (Hajighahramani et al., 2017). In addition to the single epitopes with their linkers, PADRE sequence and β-defensin adjuvant were incorporated to complete the potential multitope vaccine construct. Finally, we assessed the multitope vaccine construct for its antigenicity, allergenicity, toxicity, and percentage of population coverage through VaxiJen v2.0, AllerTop v2.0 (Dimitrov et al., 2014), ToxinPred (Gupta et al., 2013), and IEDB (Bui et al., 2006) webservers.

### Physicochemical features, protein solubility assessment, and secondary structure prediction

In this stage, we utilized ProtParam, a tool available on the Expasy server (Gasteiger et al., 2005) SOLpro server (Magnan et al., 2009), and PSIPRED 4.0 (Buchan and Jones, 2019) web server in order to anticipate the physicochemical properties, the propensity upon overexpression in *E. coli*, and the protein secondary structure of the generated potential vaccine construct from the previous step.

### Tertiary structure prediction, refinement, and validation

After the design with physicochemical and immunological properties assessment of the multitope vaccine construct, we aimed to predict its 3D structure to be used for a docking study with the human immune receptor. For this purpose, the Robetta server (Kim et al., 2004) was employed. Robetta server utilizes a unique approach for protein structure prediction where if a confident match to a protein of known structure is found using BLAST, PSI-BLAST, FFAS03, or 3D-Jury, this protein is employed for the modeling process. Alternatively, if no match is found, the modeling process is performed through the *de novo* Rosetta fragment insertion method. Following that, we utilized the GalaxyRefine server (Heo et al., 2013) to refine the 3D protein structure estimated by Robetta and evaluated this refinement through the generated scores of Ramachandran plot analysis (Laskowski et al., 1993) and ProSA (Wiederstein and Sippl, 2007).

### Conformational B-cell epitope prediction

While continuous B cell epitopes, which were predicted in the above sections, are estimated through the primary amino acid sequence of a protein, another type of epitopes, which is conformational (or discontinuous) B cell epitopes, are predicted based on the 3D structure of the antigenic protein. For this purpose, the ElliPro Server (http://tools.iedb.org/ellipro) was utilized.

### Molecular docking-coupled dynamics simulations

Prior to molecular docking simulations, proteins such as the human TLR2 (PDB; 2Z7X) and TLR4 (PDB: 3FXI) and constructed hybrid multi-epitope vaccine were independently prepared through partial charge assignment, 3D-protein protonation and/or removal of any bound ligand(s), crystalized solvent, and ionic metals/salts (Helal et al., 2020; Elhady et al., 2021; Elmaaty et al., 2022). For identifying the active and surrounding residues within the structures of the investigated proteins, the vaccine and TLR targets were submitted at Consensus Prediction Of interface Residues in Transient complexes (CPORT; https://alcazar.science.uu.nl/services/CPORT/) (de Vries and Bonvin, 2011). Docking the constructed vaccine on the TLRs was performed using an on-line docking server; ClusPro v2.0 (Boston and Brook Universities; https://cluspro.org/) (Kozakov et al., 2017; Porter et al., 2017). Relying on the Fast Fourier Transform correlation protocol, ClusPro predicted the vaccine/TLR4 complex through a multi-stage process; PIPER-based rigid docking, interaction energy-based conformation filtration, ranking based on clustering properties, and finally refinement through minimization (Kozakov et al., 2006). Interaction energy adopted by ClusPro included energy terms for van der Waals (EVDW = Eatt + Erep), electrostatic (Eelec), and pairwise structure-dependent potentials (EDARS) resulting from Decoys as reference state, however, lacked entropic energy terms (Chuang et al., 2008). It was suggested to utilize the cluster ranking, in terms of cluster populations, rather than the obtained ClusPro interaction energy for ranking and identifying the best-clustered structure complex (Zhang et al., 2022).

Evaluation of the best docking pose proceeded using the online PDBePISA v1.5.2 server tool (European Bioinformatics Institute/EMBL-EBI; https://www.ebi.ac.uk/msd-srv/prot_int/cgi-bin/piserver) for macromolecular interface analysis (Evgeny, 2010; Schlee et al., 2019). This tool provided descriptions for the sole and bound protein interface including interface residues, total solvent-accessible surface area (Å2), numbers/types of binding interactions, as well as the gained solvation-free energy (ΔiG; Kcal/mol), and its *p*-value (ΔiG *p*-value). The last two descriptors are indices for higher interface hydrophobicity/protein affinity (high negative ΔiG values) and to how many degrees the protein-protein interface can be interaction-specific (*p* < 0.5) (Krissinel and Henrick, 2007). Estimating the vaccine binding affinity for the predicted docked complex was further investigated using the Molecular Mechanics energy-guided Generalized Born and Surface Area (MM/GBSA; Kcal/mol) calculations implemented at the HawkDock server (Zhejiang University; http://cadd.zju.edu.cn/hawkdock/) (Weng et al., 2019). HawkDock MM/GBSA calculation permitted an estimation of energy term components including van der Waal, electrostatic, Generalized Born-predicted polar solvation free energy, and empirical model-predicted non-polar solvation contribution, besides dissection them down to the protein’s per-residue energy contributions (Hou et al., 2011; Zhong et al., 2020).

The best-docked vaccine-TLR complex was subjected to 100 ns all-atom molecular dynamics simulations under CHARMM36m forcefield and GROMACS program (Páll et al., 2015). Protein complexes were solvated at TIP3P cubic box under periodic boundary conditions maintaining a minimum distance of 10 Å between the protein atoms and box boundaries. The net charge of the system was neutralized *via* sufficient 0.15 M sodium and chloride ions. Systems were subjected to the steepest-descent minimization at 0.05 ns, followed by two-staged equilibration at standard thermo- and barostats (Berendsen-temp for NVT ensembles; 1 ns at 310 K followed by Parrinello-Rahmann barostat for NPT ensemble; 1 ns at 1 atm. and 310 K. Molecular dynamics were run for 100 ns under NPT ensemble and Particle-Mesh-Ewald algorithms for computing long-range electrostatic interaction (Andricioaei and Karplus, 2001). Trajectory analysis was performed using root-mean-square deviations (RMSDs; Å), radii of gyration (ROG; Å), solvent-accessible surface area (SASA; Å^2^), and RMS-fluctuations (RMSFs; Å) relying on the protein’s backbone alpha-carbon atoms. Conformational analysis and visualization of the simulated complexes at specified timeframes were done using PyMOL software (Schrödinger; v2.0.6, United States).

### Immune simulation of the designed vaccine

The final stage of our study was the prediction of the generated immune response upon the administration of the potential vaccine construct where The C-ImmSim server (Rapin et al., 2010) was employed for this purpose. In the estimation, we utilized the prime-booster-booster technique, an approach that was carried out *via* the injection of the potential vaccine three times at 4 weeks intervals. This approach has been followed to get a long-lasting immune response.

### Reverse translation and codon optimization

The last stage of the current study was the codon optimization for the designed potential vaccine where we employed the JCAT server for this purpose (Grote et al., 2005). Here, we selected *E. coli* k-12 strain as the expression organism as it is frequently used in gene cloning experiments (the first stage for wet lab validation of the current potential vaccine). The codon adaptation index (CAI), a value that is calculated by the server, gives an estimation for the constructed potential vaccine to be expressed in *E. coli* k-12.

## Results

### Selection of vaccine candidates

Regarding *K. pneumoniae*, 9 protein candidates namely FepA, FepB, FepC, FhuA, FhuF, FuR (iron uptake proteins), OmpA, OmpC, and OmpF (outer membrane proteins) were filtered through their antigenicity score estimated by VaxiJen v2.0 with the cutoff score of 0.4 (the threshold value of bacterial antigenic proteins) and the final 2 protein candidates of *K. pneumoniae* were FepA and OmpF with antigenicity scores of 0.76 and 0.81 respectively (highest scores). Moving to *P. aeruginosa*, the same approach was followed where six protein candidates namely FoxA, FpvA, HasR, HitA (iron uptake proteins), OprF, and OprH (outer membrane proteins) were filtered and 2 proteins (also one from each category) namely HasR and OprF with the antigenicity scores of 0.59 and 0.8 respectively were selected as our final candidates for *P. aeruginosa*. Collectively, 4 proteins were selected as final targets for epitope prediction (2 proteins per microorganism).

### T and B cell epitopes selection

At this stage, the 4 filtered protein candidates were mapped for their T and B cell epitopes. For T cell epitopes, the mapping of MHC-I epitopes resulted in a large number of epitopes for each protein with a percentile rank ranging from 0.01 to 100 where a small percentile rank represents a better binding in comparison to the large percentile rank score, and for this reason, we filtered only the epitopes with percentile score less than 1 (to guarantee for good binding) and the best candidates with a large antigenicity score were listed in Table 1 (that demonstrates the top five epitopes identified for each protein). In addition to that, the same 4 protein candidates were mapped for MHC-II epitopes and the output results were arranged based on the binding affinity. For this type of epitope, we selected only the top 1% of the results and selected the best candidates based on antigenicity score and the ability of the epitope to induce INF-γ, IL-4, IL-10, IL-6, and IL-13 cytokines. Table 2 combines the top five epitopes identified for each of the 4 selected proteins. Lastly, we mapped for B cell epitopes using the BepiPred-2.0 prediction method where the peptides with a length between 9:20 amino acids were submitted to VaxiJen for antigenicity score estimation (Table 3) and the ones that were highly antigenic were selected for the multitope vaccine construction.

**TABLE 1:** A list of filtered top-ranked T-cell epitopes (MHC-I peptides) of FepA, OmpF, HasR, and OprF proteins.

No	Protein	Start-end	Epitope	Antigenicity
1	FepA	625–633	KQEPKKYNY	1.17
2	FepA	614–622	VSLQSTFTW	0.87
3	FepA	600–608	YTLNSTLSW	0.6
4	FepA	468–476	QTNPNYILY	1.03
5	FepA	499–507	AETSINKEI	0.9
6	OmpF	126–134	RTNGVATYR	1.05
7	OmpF	46–54	QINDQLIGY	0.81
8	OmpF	34–42	TTYARIGLK	1.07
9	OmpF	189–197	SSNRSVDQK	1.9
10	OmpF	28–36	DTSSDDTTY	1.65
11	HasR	427–435	AQAQNTSTF	0.75
12	HasR	172–180	SVDGMRQNY	1.34
13	HasR	34–42	AEQAGVQVF	0.85
14	HasR	531–539	RQTDMPLQY	0.74
15	HasR	309–317	RVKHSPVAY	0.99
16	OprF	63–71	KVHGNLTSL	1.06
17	OprF	26–35	ADLYGGSIGY	0.82
18	OprF	113–121	ANIGAGLKY	0.88
19	OprF	6–15	VEIEAFGKRY	0.67
20	OprF	224–232	KSKVKENSY	0.82

**TABLE 2:** A list of filtered top-ranked T-cell epitopes (MHC-II peptides) of FepA, OmpF, HasR, and OprF proteins.

No	Protein	Epitope	Antigenicity	IFN-γ inducer	IL4 inducer	IL10 inducer	IL6 inducer	IL13 inducer
1	FepA	IIPEYTLNSTLSWQV	1.21	Yes	Yes	No	Yes	Yes
2	FepA	GLVRWEFAPMQSLEF	0.99	Yes	No	Yes	Yes	No
3	FepA	LVRWEFAPMQSLEFE	1.46	Yes	Yes	Yes	Yes	Yes
4	FepA	VSIPFDYLVNQNLTL	0.97	No	No	Yes	Yes	No
5	FepA	FDYLVNQNLTLGSEW	0.87	Yes	No	Yes	Yes	Yes
6	OmpF	FFGLVDGLSFALQYQ	0.84	Yes	No	Yes	Yes	No
7	OmpF	AVVQYQFDFGLRPSI	1.69	Yes	Yes	Yes	Yes	Yes
8	OmpF	QNFEAVVQYQFDFGL	1.11	Yes	Yes	Yes	Yes	Yes
9	OmpF	GDGFSTAATYAFDNG	0.52	Yes	No	No	No	Yes
10	OmpF	DFFGLVDGLSFALQY	0.78	Yes	No	Yes	Yes	No
11	HasR	VSQDDLVQMSPSVIS	0.67	Yes	Yes	Yes	No	Yes
12	HasR	DLSTLRANYGLEFFY	0.94	Yes	Yes	Yes	Yes	No
13	HasR	EEGRFSPTFGLSVKP	1.6	No	Yes	No	No	No
14	HasR	ALRRVRLDIPAQPLN	0.66	No	Yes	No	Yes	Yes
15	HasR	YGSYRVSDELTLRLA	1.11	Yes	Yes	No	Yes	Yes
16	OprF	GAGLKYYFTENFFAK	0.93	Yes	Yes	No	No	Yes
17	OprF	GVGLRPYVSAGLAHQ	0.99	Yes	No	Yes	Yes	No
18	OprF	VGLRPYVSAGLAHQN	0.71	Yes	No	Yes	Yes	Yes
19	OprF	IGAGLKYYFTENFFA	0.62	Yes	Yes	No	No	Yes
20	OprF	YYFTENFFAKASLDG	0.54	No	No	No	No	Yes

**TABLE 3:** A list of predicted B cell epitopes of FepA, OmpF, HasR, and OprF proteins.

FepA			OmpF		
Epitope	Start-end	Antigenicity score	Epitope	Start-end	Antigenicity score
EQNLQAPGVST	22–32	0.9	VWTTNGDTSSDDTT	22–35	1.76
DEIRKRPPAR	36–45	1.12	DASNVEGSQTTK	62–73	2.12
GNSTSGQRGNN	61–71	2.9	QGKNDHDRAIRKQNGD	152–167	1.45
TGDEWHGSW	151–159	0.33	SNRSVDQKADGNGDKA	190–205	2.26
APEHKDEGSTKRT	165–177	1.9	TYNMTPEEDNHFAGKTQ	228–244	0.77
WNGAWDNGVTTS	295–306	0.72	TKGQGPAVA	267–275	1.35
HHSIVGNNW	429–437	1.24			
YAPVYQNNKGTDLYQW	535–550	0.81			
LQSKNKETGDRLSII	583–597	1.11			
HasR			OprF		
Epitope	Start-End	Antigenicity Score	Epitope	Start-End	Antigenicity Score
DSASQQQTALRRVRLDI	5–21	1.2	RYFTDSVRNMKNA	14–26	−0.56
QRFAGLGSAAVHGEYL	46–61	0.31	GEYHDVRGTYETGNKK	48–63	1.48
DWVYQTPHSVSV	110–121	0.54	NITNINSDSQGRQQMT	96–111	1.33
REQIERNPPRH	124–134	0.50	SVGTDAYNQKLSER	258–271	1.6
SSVSQQDPG	147–155	1.06	YGESRPVADNATAEGRA	296–312	0.95
YQQSGHQQRNGTLYVDPE	180–197	0.84			
EARDLVRPGKQVGG	228–241	0.007			
SGLGGDANGT	247–256	2.82			
YGYAPDNPLV	381–390	0.4			
FALDDLSTL	435–443	−0.22			
KENLWFSDD	617–625	−1.04			
MGMGMQPPGYGMAGIGNSA	644–662	0.78			
RFFDRRLDVG	761–770	0.93			
LVPLGDVLAFTL	830–841	0.09			

### Multitope vaccine construction and assessment for its characteristics

As the next stage after epitope mapping, we planned to select the top 2 single epitopes (for FepA, OmpF, HasR, and OprF) from our final list of MHC-I, MHC-II, and B cell epitopes and after selecting the most promising B and T cell epitopes, we get a final count of 8 CTL, 8 HTL, and 8 BCL epitopes. These epitopes were linked together using GGGS, GPGPG, and KK linkers respectively. Following that, 2 important components, namely PADRE peptide and β-defensin, were incorporated to finalize the sequence of the multitope construct which lastly constituted 476 amino acids and sequenced as the following:

“EAAAKGIINTLQKYYCRVRGGRCAVLSCLPKEEQIGKCSTRGRKCCRRKKEAAAKAKFVAAWTLKAAAGGGSKQEPKKYNYGGGSQTNPNYILYGGGSRTNGVATYRGGGSDTSSDDTTYGGGSSVDGMRQNYGGGSRVKHSPVAYGGGSKVHGNLTSLGGGSKSKVKENSYGPGPGLVRWEFAPMQSLEFEGPGPGIIPEYTLNSTLSWQVGPGPGFFGLVDGLSFALQYQGPGPGAVVQYQFDFGLRPSIGPGPGVSQDDLVQMSPSVISGPGPGDLSTLRANYGLEFFYGPGPGGAGLKYYFTENFFAKGPGPGGVGLRPYVSAGLAHQKKGNSTSGQRGNNKKAPEHKDEGSTKRTKKDASNVEGSQTTKKKSNRSVDQKADGNGDKAKKDSASQQQTALRRVRLDIKKSGLGGDANGTKKNITNINSDSQGRQQMTKKSVGTDAYNQKLSERKKAKFVAAWTLKAAAGGGS”.

As mentioned in the methodology section, T cell epitopes mapping was performed against the HLA allele reference set to achieve a high percentage of population coverage, consequently, assessment of the percentage of population coverage revealed the scores of 92.56%, 99.98%, and 100% for world MHCI, MHCII, and combined population coverage respectively (Supplementary Figure S1). Prior to the tertiary structure prediction and analysis of the docking properties of the potential vaccine, it was essential to test and assess its physicochemical properties. The potential vaccine sequence was submitted to AllerTop, VaxiJen, and ToxinPred servers to assess for its allergenicity, antigenicity, and toxicity and the server’s output revealed that our potential vaccine is non-allergen, non-toxic, and antigenic with an antigenicity score of 1.38. Moreover, the potential vaccine sequence was analyzed for its solubility upon overexpression and obtained a score of 0.85 which was a satisfactory one (a score that exceeds 0.5 indicating solubility upon overexpression). Another essential analysis for the potential vaccine sequence was the assessment through Blastp where the results demonstrated that the submitted multitope sequence did not significantly resemble human protein sequences therefore this potential vaccine would not elicit autoimmune reactions during human usage. Furthermore, ProtParam online tools were employed to assess the multitope construct for its other physicochemical properties where the satisfactory output scores were summarized in Supplementary Table S1. Lastly, the secondary structure prediction for the multitope construct was performed using PSIPRED 4.0 webserver and the output predicted 28% helix, 13.4% strand, and 58.6% coil of the potential vaccine construct secondary structure (Supplementary Figure S2).

### Tertiary structure prediction, validation, and refinement

**FIGURE 2:**
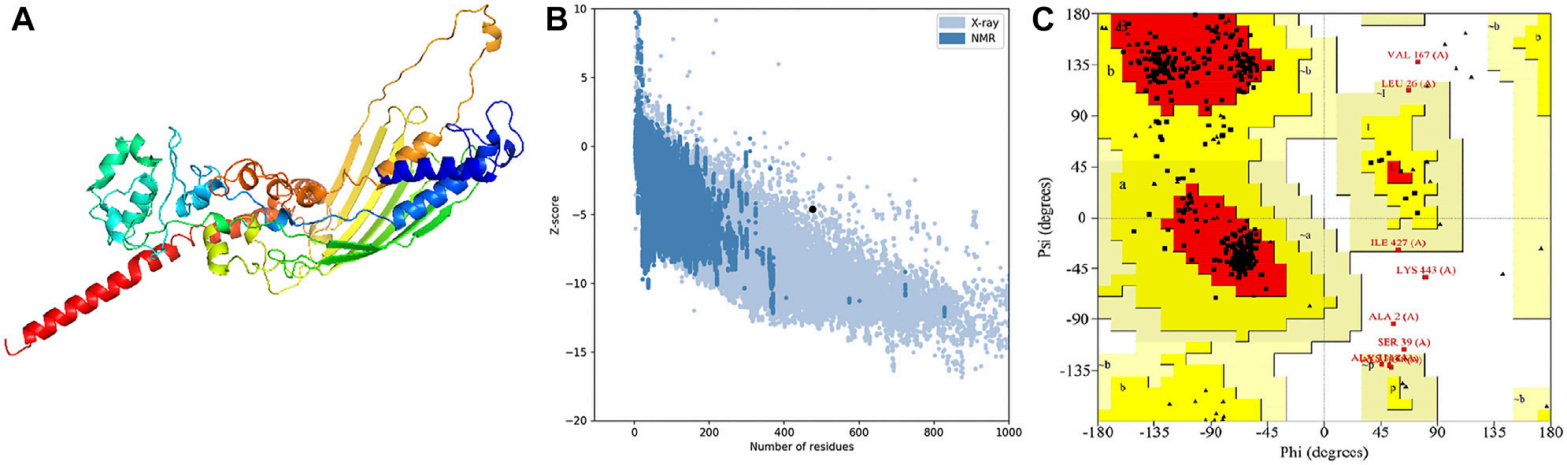
Structural analysis of the multitope vaccine tertiary structure. **(A)** The 3D structure of the designed vaccine after refinement; **(B)** ProSA evaluation of the refined vaccine where the black dot shows the exact Z-score; **(C)** Ramachandran plot assessment for the refined vaccine construct.

In order to assess the chemical properties of the potential multitope vaccine it was essential to predict its tertiary structure and for this purpose, we employed the Robetta webserver. The generated 3D model was then uploaded to the GlaxyRefine webserver where Ramachan plot analysis and ProSa Z-score were utilized to assess the quality of the original model generated by Robetta and the refined one. For the current study primary model (output of Robetta prediction), 85.3%, 13.4%, and 1.4% of residues were located in favored, allowed, and outlier regions, respectively. After refinement on the GlaxyRefine server, the refined model demonstrated 89.9%, 10%, and 1.1% of residues located in favored, allowed, and outlier regions, respectively with a Z-score of −4.59. The predicted tertiary structure of the multitope construct and the output of its structural validation are shown in Figure 2.

### Conformational B-cell epitope prediction

**FIGURE 3:**
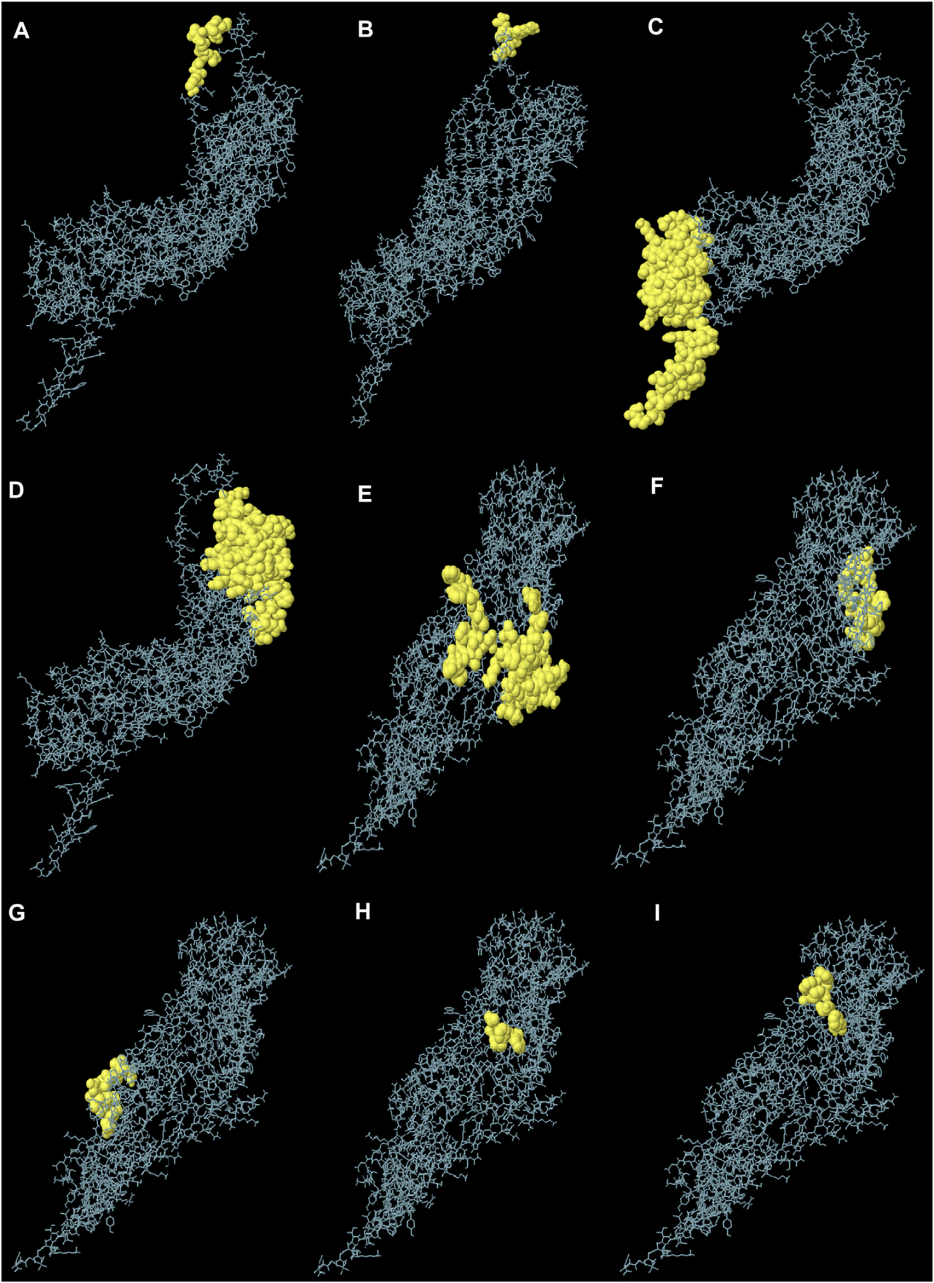
Predicted conformational B cell epitopes. Each symbol represents one discontinuous B cell epitope **(A–I)**, in agreement with the data in Table 4.

The generated 3D structure of the multitope construct was uploaded to EIIiPro webserver for the prediction of conformational B cell epitopes. The server estimated 9 discontinuous epitopes (Figure 3) with a score ranging between 0.578 and 0.977. Table 4 mentions the residues of each predicted epitope and the corresponding score.

**TABLE 4:** Conformational B cell epitopes residues of the potential vaccine construct as predicted by ElliPro.

Epitope symbol	Residues	Number of residues	Score
A	A:K334, A:G335, A:N336, A:S337, A:T338, A:S339, A:G340, A:Q341	8	0.977
B	A:R342, A:G343, A:N344, A:N345, A:K346	5	0.975
C	A:G82, A:G83, A:G84, A:G96, A:G97, A:S98, A:R99, A:T100, A:N101, A:G102, A:V103, A:A104, A:T105, A:Y106, A:R107, A:G108, A:G109, A:D112, A:T113, A:S114, A:S115, A:D116, A:D117, A:T118, A:T119, A:Y120, A:G121, A:G122, A:G123, A:S124, A:S125, A:V126, A:D127, A:G128, A:M129, A:R130, A:N132, A:Y133, A:G134, A:G135, A:G136, A:S137, A:R138, A:V139, A:K140, A:H141, A:S142, A:P143, A:V144, A:A145, A:Y146, A:G147, A:G148, A:G149, A:S150, A:K151, A:V152, A:H153, A:G154, A:N155, A:L156, A:T157, A:S158, A:L159, A:G160, A:G161, A:G162, A:S163, A:K164, A:S165, A:K166, A:V167, A:L454, A:S455, A:E456, A:R457, A:K458, A:K459, A:A460, A:K461, A:F462, A:V463, A:A464, A:A465, A:W466, A:T467, A:L468, A:K469, A:A470, A:A471, A:A472, A:G473, A:G474, A:G475, A:S476	95	0.728
D	A:T10, A:L11, A:K13, A:Y14, A:Y15, A:C16, A:R17, A:V18, A:R19, A:G20, A:G21, A:R22, A:C23, A:A24, A:V25, A:L26, A:S27, A:C28, A:L29, A:P30, A:K31, A:E32, A:E33, A:Q34, A:I35, A:G36, A:K37, A:C38, A:S39, A:T40, A:R41, A:G42, A:R43, A:K44, A:C45, A:C46, A:R47, A:R48, A:E51, A:Q187, A:S188, A:L189, A:F191, A:E192, A:G193, A:P194, A:G195, A:P196, A:G197, A:I198, A:I199, A:P200, A:Y202, A:L204, A:Y231, A:G233, A:P234, A:G235, A:K347, A:A348, A:P349, A:E350, A:H351, A:K352	64	0.724
E	A:W181, A:W210, A:D223, A:Y242, A:L248, A:P250, A:S251, A:P274, A:G275, A:P276, A:G277, A:D278, A:L279, A:S280, A:L282, A:Y286, A:E307, A:F309, A:A311, A:K312, A:G313, A:P314, A:G315, A:P316, A:G317, A:V319, A:L321, A:P323, A:S414, A:G415, A:L416, A:G417, A:G418, A:D419, A:A420, A:N421, A:G422, A:T423, A:K424, A:K425, A:N426, A:I427, A:T428, A:N429	44	0.619
F	A:Y292, A:G293, A:P294, A:G295, A:P296, A:G297, A:G298, A:A299, A:L301, A:Y303, A:V325, A:S326, A:A327, A:G328, A:L329, A:A330, A:H331, A:D353	18	0.604
G	A:Y81, A:S380, A:V381, A:D382, A:Q383, A:K384, A:A385, A:D386, A:G387, A:N388, A:G389	11	0.604
H	A:L225, A:F244, A:F246	3	0.58
I	A:F58, A:W62, A:P185, A:S206, A:F227	5	0.578

### Molecular docking and binding pose prediction

**FIGURE 4:**
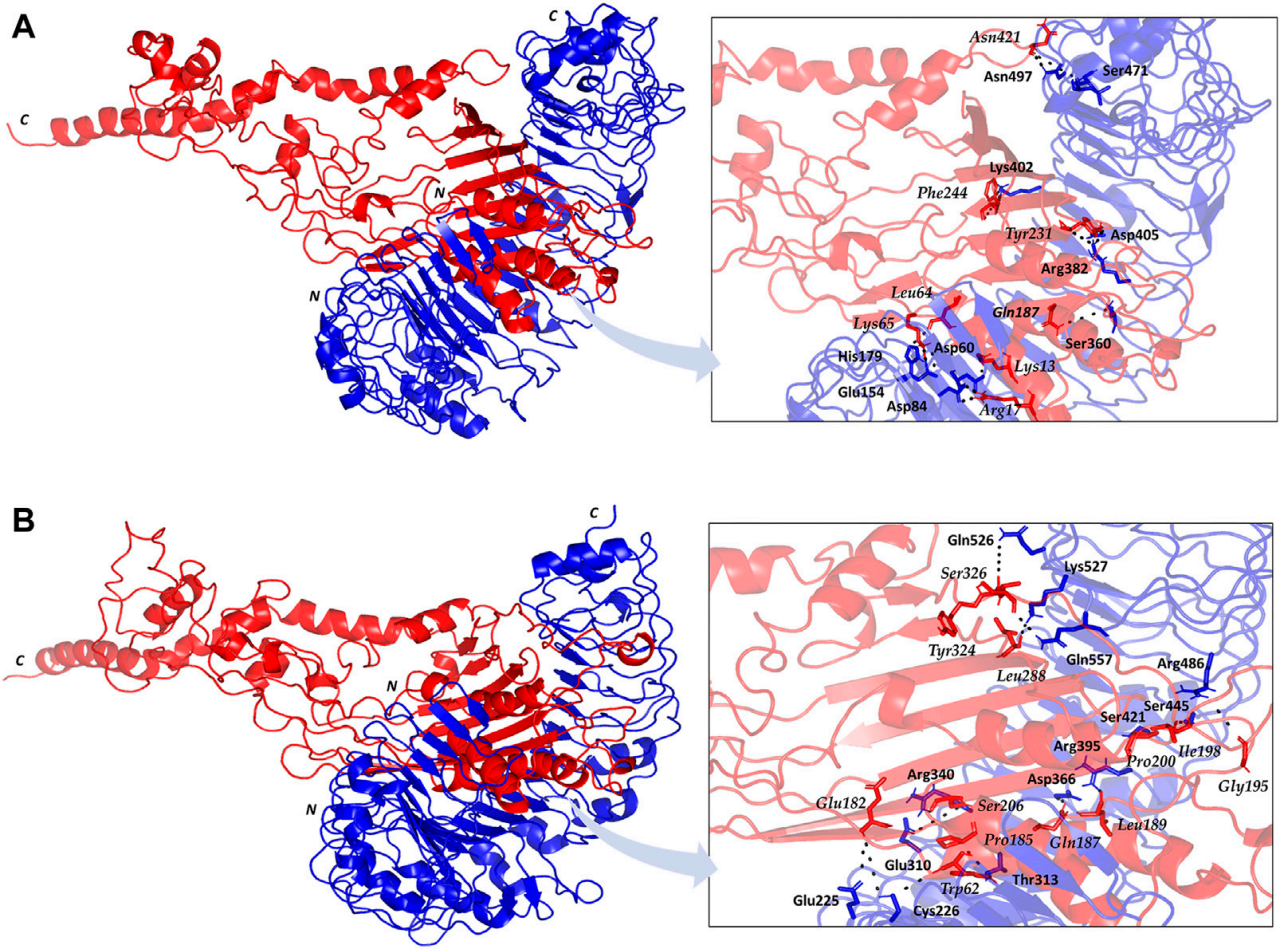
Cartoon representation for docked vaccine-TLR complex obtained from ClusPro server **(A)** TLR4; **(B)** TLR2. Multiepitope vaccine (red) anchored at the inner concave interface of TLR (blue). Bold letters *N* and *C*; denote the protein’s amino and carboxy terminals, respectively. Residues mediating vaccine-TLR hydrophilic binding interactions are labeled based on their sequence and colored with respect to their location. Zoomed images illustrate the vaccine-TLR binding interface with polar hydrogen bonding being represented as black dashed lines.

The employed docking server illustrated significant binding of the constructed multiepitope vaccine at both TLR isotypes, TLR4 and TLR2, where these innate immunity receptors are highly reported for their important role within the host’s defense throughout infections by *K. pneumonia* (Wieland et al., 2011; Paczosa and Mecsas, 2016; Jeon et al., 2017; Dar et al., 2019; Allemailem, 2021). Selection of the relevant binding mode was guided by furnishing high docking scores as well as achieving contacts with the CPORT-suggested hot-spot residues for relevant protein-protein binding (Supplementary Figure S3). Binding modes were quite comparable at both TLRs, where the vaccine was anchored at the inner concave surface of the TLRs by the virtue of the latter extended conformation shaping as a paddle (Figure 4). Relevant vaccine/TLR binding was mediated by the vaccine’s anti-parallel β-sheets near its *N*-terminus. Notably, the vaccine *N*-terminus was further extended towards the other side, yet it was kept at relatively small distances. Representing the handle of a paddle, the α-helices at the vaccine’s carboxy end depicted extended conformation towards the solvent side being quite far from the TLR binding interface. Analyzing the complex interfaces *via* PDBePISA illustrated a relatively larger interface solvent-accessible area as well as interaction contacts (combined hydrogen bonds and salt bridges) for the obtained vaccine towards TLR4 over those at TLR2 (Table 5). Anchoring preferentiality at TLR4 was also translated into higher negative Δ1G scores as well as lower Δ1G *p*-value implying profound interaction-specificity for the vaccine and TLR4 interface surface being of higher hydrophobicity than would be averaged for given structures. Based on the above findings, the obtained vaccine-TLR complexes were considered significant for further interface evaluation and to be used for subsequent molecular dynamic simulation studies.

**TABLE 5:** Descriptors of the multiepitope vaccine/TLRs interface analysis predicted *via* the PDBePISA server.

Target receptor			Multiepitope vaccine		Interface				
Isotype	Interface	Interface	Interface	Interface	Interface	№. H-bonds	№. Salt bridges	Δ^i^G^c^ (Kcal/mol)	Δ^i^G*P*-value^d^
	Residues	Surface^a^ (Å^2^)	Residues	Surface^a^ (Å^2^)	Surface^b^ (Å^2^)				
TLR4	89	23,768	76	23,789	2,678	12	8	−34.1	0.091
TLR2	91	23,489	81	23,779	2,217	14	0	−29.2	0.188

^a^ Solvent-accessible surface area within squared angstrom units for each bound protein.

^b^ Interface area denotes the difference in accessible surface areas of isolated and interfacing structures divided by two.

^c^ Δ^1^G denotes the gained solvation-free energies through interface formation. Higher negative values imply hydrophobic interfaces and positive protein affinity.

^d^ Δ^1^G *p*-value denotes the *p*-value of the gained solvation-free energies. It measures the probability of getting a lower Δ^1^G than the observed Δ^1^G when the interface atoms are randomly picked from the protein’s surface. For Δ^1^G *p*-values below 0.50 imply that the interfaces of surprising hydrophobicity, even higher than the would-be-average for given structures, the thing that further implies that the interface surface could be interaction-specific.

Key binding residues at the interface between the vaccine and each TLR target were also highlighted *via* PDBePISA interface analysis (Table 6; vaccine residue will be represented in italics starting from here forward). At the TLR4 bound complex, the vaccine *Arg17* was depicted as significant for mediating highly network polar contacts, both hydrogen bonds, and salt bridges, with the corresponding TLR4 interface anionic residues (aspartates; Asp60 and Asp84) (Figure 4A). Additionally, the cationic *Lys13* and *Lys65* at the vaccine’s loop region mediated both hydrogen bond and salt bridge with the respective carboxylate sidechain of Glu154 and Asp60 at the TLR4 side. Several other polar vaccine residues including; *Tyr231*, *Gln187*, *Asn421,* as well as the mainchain of *Phe244* predicted relevant hydrophilic contacts with TLR4 amino acids at distinct proximal distances (1.19 to 3.48 Å). Besides polar interacting residues, hydrophobic interface residues of the multiepitope vaccine showed significant closeness and relevant non-polar contacts with neighboring TLR4 amino acids. Vaccine hydrophobic residues including; Trp181, Phe183, Tyr202, Leu208, Phe227, Tyr231, Tyr242, Phe244, Tyr286, Leu288, Tyr292, Tyr303, Phe305 depicted ≤5.0 Å distances from Met41, His229, His256, Phe377, Tyr403, Tyr451, His426, Ile450, Phe408, Phe500, and/or Val602 of the TLR4 interface, respectively.

**TABLE 6:** Key interface per-residue polar interactions *via* PDBePISA and predicted MM/GBSA binding energies.

Target receptor	Polar interactions		MM/GBSA calculations (Kcal/mol)		
	Hydrogen bonds	Salt bridges	Total binding energy	Per-residue contributions^a^ (≥2.00 Kcal/mol)	
				TLR	Vaccine
TLR4	His179[NH]: *Leu64*[O] 1.77 Å	Asp60[OD1]: *Lys13*[NH1] 3.76 Å	−198.21	Asp60	*Arg17*
	Arg382[HH21]: *Tyr231*[OH] 1.96 Å	Asp60[OD2]: Lys*13*[NZ] 3.06 Å		Asp84	*Tyr231*
	Asp84[OD1]: *Arg17*[HH12] 1.28 Å	Asp60[OD1]: *Arg17*[NH1] 3.12 Å		Glu154	*Phe227*
	Asp84[OD2]: *Arg17*[HH22] 1.49	Asp60[OD2]: *Arg17*[NZ] 3.43 Å		Phe377	*Trp181*
	Glu154[OE1]: *Lys65*[HZ1] 1.16 Å	Asp84[OD1]: *Arg17*[NH2] 3.42 Å		Ile450	*Tyr286*
	Ser360[OG]: *Gln187*[HE21] 2.49 Å	Asp84[OD2]: *Arg17*[NZ] 2.50 Å		Tyr451	*Phe183*
	Asn497[OD1]: *Asn421*[H] 1.88 Å	Glu154[OE2]: *Lys65*[NH1] 3.77 Å		His426	*Tyr242*
	Ser471[O]: *Asn421*[HD21] 3.36 Å	Glu154[OE1]: *Lys65*[NZ] 2.47 Å		Asp405	*Phe244*
	Lys402[NH2]: *Phe244*[O] 2.47 Å			Ser472	*Leu288*
	Lys402[NH1]: *Phe*244[O] 2.48 Å			Arg382	*Asn421*
	Asp405[OD1]: *Tyr231*[OH] 2.19 Å			Phe500	*Phe305*
	Arg382[HH21]: *Tyr231*[O] 1.19 Å			Lys402	*Tyr202*
				His229	*Leu208*
				Phe573	*Leu301*
				Asp453	*Tyr292*
				Met41	*Leu204*
				Phe63	*Pro316*
				Asp428	*Leu229*
				Phe408	
				Glu79	
				Glu603	
				Glu42	
				Asn497	
				Glu31	
TRL2	Cys226[SH]: *Trp62*[O] 3.81 Å		−127.72	Cys226	*Glu182*
	Cys226[SH]: *Glu182*[O] 3.52 Å			Asp366	*Gln187*
	Thr313[OH]: *Pro185*[O] 2.26 Å			Glu310	*Trp62*
	Arg395[HH12]: *Leu189*[O] 2.32 Å			Tyr364	*Phe191*
	Arg486[HH21]: *Gly195*[O] 3.44 Å			Arg395	*Ser326*
	Ser445[OH]: *Ile198*[O] 1.52 Å			Asp463	*Ile198*
	Ser421[OH]: *Pro200*[O] 1.43 Å			Leu280	*Ser206*
	Arg340[HH21]: *Ser206*[O] 1.65 Å			Tyr483	*Phe58*
	Lys527[HZ1]: *Leu288*[O] 1.43 Å			Arg486	*Tyr286*
	Gln557[HE22]: *Ser326*[O] 1.71 Å			Glu281	*Leu288*
	Glu225[OE1]: *Glu182*[H] 2.91 Å			Tyr440	*Pro200*
	Asp366[OD1]: *Gln187*[HE21] 2.14 Å			Ser445	*Phe305*
	Glu310[ OE1]: *Ser206*[OH] 2.85 Å			Glu177	*Trp181*
	Gln526[OE1]: *Tyr324*[NH] 3.08 Å			Gln557	Thr207
				Glu228	*Leu189*
				Glu225	*Pro185*
				Lys527	*Phe227*
				Asp285	*Gly195*
				Glu344	*Val240*
				Ser421	*Val325*
				His202	*Tyr324*
				Gln526	*Ser326*
				Asp286	*Gln187*
				Arg340	*Phe246*
				Glu103	*Pro185*
				Ser445	*Leu225*
				Thr313	*Ser206*
				Asp106	
				Tyr109 Asp58	

^a^ Per-residue MM/GBSA energy contribution being listed in descending order.

Moving towards the vaccine-TLR2 docking complex, several polar contacts were depicted majorly through hydrogen bond contacts without relevant salt bridges (Figure 4B). The vaccine’s acidic residue, *Glu182*, was the most frequent amino acid contributing to the vaccine’s parallel β-sheet stability towards the TLR2 concave interface. Other polar vaccine residues mediated significant hydrogen bonding with corresponding TLR2 amino acids at close distances and relevant angles (1.65 to 2.35 Å and 141 to 179 Å). Interestingly, the peptide backbones of several vaccine’s hydrophobic residues including; *Trp62*, *Pro185, Leu189, Gly195, Ile198, Pro200, Leu288, and Tyr324,* depicted relevant polar contacts with close TLR2 residues at the binding interface. In addition to hydrophilic residue pairing, hydrophobic interface residues of the multiepitope vaccine showed significant closeness and relevant non-polar contacts with neighboring TLR2 amino acids. Vaccine’s hydrophobic residues such as; *Phe58*, *Val59*, *Trp181*, *Phe183*, *Met186*, *Phe191*, *Ile199*, *Leu208*, *Trp210*, *Leu225*, *Phe227*, *Leu229*, *Ala238*, *Val240*, *Tyr242*, *Phe246*, *Tyr286*, *Phe290*, *Tyr292*, *Leu301*, *Tyr302*, *Phe305*, *Val325*, *Ala327*, and *Leu329* showed distances less than 5.0 Å from Tyr109, His202, Ile204, Leu250, Ile251, Leu280, Tyr364, Leu392, Ile393, Tyr440, Ile461, Tyr483, Met500, Leu502, Val503, Trp529, His 531, and/or Pro568 of the TLR4 side.

Putting all the above preferential per-residue interaction within energy terms, the MM/GBSA binding energy calculations were estimated for both docked vaccine/TLR complexes. As expected, higher residue-wise energy contributions were assigned for the key interacting residues of both vaccine and TLR proteins (Table 6). The higher negative total binding energy has been correlated to the preferential vaccine affinity towards the TLR4 interface (−198.21 Kcal/mol) as compared to that of TLR2 (−127.72 Kcal/mol). Electrostatic energy contributions (−8,323.49 Kcal/mol and −5,374.32 Kcal/mol) were higher than those of hydrophobic van der Waal potentials (−270.25 Kcal/mol and −251.28 Kcal/mol) for TLR4 and TLR2 complexes, respectively. A penalty for polar solvation-free energy was estimated at 8,431.56 Kcal/mol, while a non-polar contribution at −35.93 Kcal/mol corresponds to a relatively large TLR4 hydrophobic surface available for vaccine binding. A similar pattern of polar and non-polar solvation-free energies was depicted at the TLR2 interface, yet at lower values (5,528.13 Kcal/mol and −30.25 Kcal/mol) than those at TLR4. The electrostatic potential preferentiality was further illustrated since several key binding polar residues showed the top-five binding energy contribution values (*Arg17*–11.06 Kcal/mol, *Tyr231*–9.01, Asp60–7.18 Kcal/mol, Asp84–7.01 Kcal/mol, and Glu154–5.71 Kcal/mol) conferring their significant role for vaccine binding and TRL4-associated complex stability. Similarly, the vaccine’s polar residues; *Glu182*–8.49 Kcal/mol, *Gln187*–7.01 Kcal/mol, and *Ser326*–6.82 Kcal/mol, as well as those of TLR2 interface; Cys226–5.51 Kcal/mol, Asp366–4.28 Kcal/mol, and Glu310–3.79 Kcal/mol, were assigned with the high binding energy contributions.

### All-atom molecular dynamics simulation and thermodynamic stability

**FIGURE 5:**
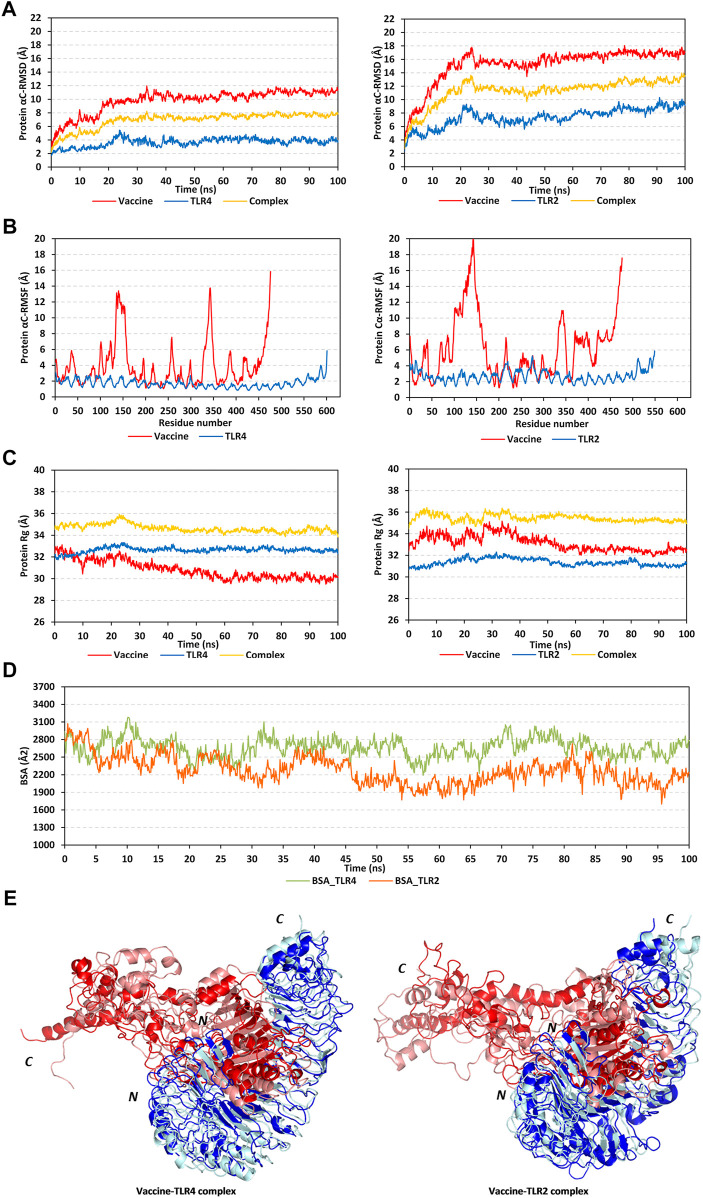
Stability analysis of the simulated multitope vaccine/TLR complex across the molecular dynamics simulations. **(A)** Protein Cα-atom RMSDs; **(B)** Protein Cα-atom RMSFs, residue range for the vaccine (*N*-terminal Glu1-to-Ser476 *C*-terminal), TLR4 (*N*-terminal Glu27-to-Cys627 *C*-terminal), and TLR2 (*N*-terminal Ser27-to-Pro575 *C*-terminal); **(C)** Protein ROGs; **(D)** Buried surface area (BSAs), as a function of the simulation times (ns). **(E)** Overlaid vaccine-TLR complex snapshots at 0 and 100 ns. Proteins are represented as cartoons and colored red and blue for respective vaccine ligand and TLR receptor. Initial and final extracted frames were represented in dark or faint colors, respectively. Bold letters *N* and *C*; denote the protein’s amino and carboxy terminals, respectively.

The RMSD trajectories of the multitope vaccine, TLR proteins, and their respective complex were monitored across the 100 ns all-atom simulation runs in reference to the alpha-carbon atoms (αC) of their respective initial structures. All monitored RMSD tones showed a gradual increase for the first 25 ns, while after that RMSDs started to attain steadier trajectories till the end of the simulation runs (Figure 5A). The latter corresponds to optimum thermodynamic behavior since all the constraints were removed with the simulation start and the proteins began to relax, till they converged at their equilibration plateau having their RMSDs being fluctuated across their respective averages. General trends of higher RMSD tones and fluctuations were assigned for the vaccine/TLR2 complex in relation to that with TLR4 (6.92 ± 1.16 Å and 11.34 ± 1.83 Å for TLR4 and TLR2, respectively). Regarding the comparative TLR protomeric units, significantly lower RMSD values were depicted for TLR4 (3.65 ± 0.62 Å) than for TLR2 (7.39 ± 1.33 Å). Similarly, the vaccine in complex to TLR4 depicted steadier and less fluctuation RMSD tones (9.77 ± 1.73 Å) as compared to its RMSD tones in complex with TLR2 (15.23 ± 2.56 Å). It is worth noting that the monitored RMSDs of the vaccine/TLR complex were more influenced by the TLR thermodynamic behaviors over those of the vaccine showing values close to the receptor alpha-carbon atoms.

Dissecting the protein fluctuation pattern down to their respective residues, the αC-RMSF trajectories were monitored across the simulation run for each simulated protein, including the vaccine and TLR protomeric unit (Figure 5B). Notably, vaccine residues depicted higher fluctuation patterns as compared to their corresponding bounded TLR, the thing that was further recapitulated by the above-depicted RMSD tones. Additionally, general trends of higher RMSF values were assigned to the vaccine-TLR2 complex over those for vaccine-TLR4 ones (3.89 ± 2.80 Å/1.72 ± 0.55 Å for vaccine/TLR4 versus 6.16 ± 3.91 Å/2.73 ± 0.76 Å for vaccine/TLR4). The highest vaccine fluctuations up to 10.05–12.72 Å were assigned to its *C*-terminal residues and vicinal amino acid region (extending across Ala327-to-Pro349 at both TLR4 and TLR2 systems; Glu369 to Ala303 only at TLR2 system). On the other hand, the vaccine’s *N*-terminal residues were of relevant stability and lower RMSF values. However, single residue-range Thr100-Gly162 depicted the highest fluctuations at the TLR4-bounded system (RMSF up to 13.03 Å) and even much higher flexibility at the TLR2-bounded one (RMSF up to 19.87 Å). It is worth noting that both vaccine’s side high-fluctuating residue regions were parts of the vaccine that are greatly extended towards the solvent side, quite distal from the TLR binding interface. Regarding the inherited fluctuations of the simulated TLR proteins, RMSF tones were at regular fluctuation patterns resembling repetitive camel humps. Interestingly, typical protein dynamic behaviors were depicted for simulated TLRs since their core residue ranges were assigned with lower RMSFs than those at the terminal sides. The observed differential fluctuation patterns between the vaccine and/or TLRs would question the influence of the protein’s inherited ternary structure, dimerization interface, and vaccine binding on protein simulation patterns.

Two other trajectory-based stability parameters, ROG and SASA, were also monitored across the simulation runs. In relation to their respective central masses, both simulated multitope vaccines showed differential ROG values across different simulation times (Figure 5C). Bound to the TLR4 protein target, the vaccine’s ROG tones gradually decreased across an average of 32.50 ± 0.64 Å from the simulation start till halfway of the simulation run (∼55 ns). Afterward, the vaccine’s ROGs were maintained across a plateau of 30.13 ± 0.29 Å which was kept steady with minimal fluctuations till the end of the simulation. Similar dynamic behavior was depicted for the TLR2-bound vaccine, yet higher ROG values (33.15 ± 0.74 Å) were obtained while being accompanied by higher fluctuations, particularly at the first half of the simulation run. This was reflected in the total ROG of the vaccine-TLR2 complex showing significant fluctuations before the 50 ns frames. Notably, both described vaccine’s ROG findings conferred differential conformation changes being attained by the vaccines beyond the first 50 ns time frames. Regarding TLR data, much steadier ROG tones were depicted for the target protein as compared to those of the bound vaccines owing to the TLR inherited stability and higher central masses. Limited ROG fluctuations were depicted for TLR4 in relation to those TLR2 (32.65 ± 0.25 Å versus 31.37 ± 0.33 Å, respectively). Complex ROGs were maintained steady over an average of 43.64 ± 0.36 Å for TLR4 and 35.45 ± 0.41 Å for TLR2, with relevant fluctuations below 50 ns time frames. It is worth noting that ROG data should be carefully interpreted since higher ROG of TLR4 and related complex are more reasoned for the larger size (more constituting residues) of TLR4 over TLR2, rather than just reflecting structure compactness and stability (Tanner, 2016).

Regarding the SASA trajectory-based analysis, buried surface area (BSA; Å^2^) for each simulated complex was estimated by adopting the sole SASAs of each bounded protein (SASA_vaccine_ and SASA_TLR_) in addition to the entire bounded complexes; BSA = 0.5*(SASA_vaccine_ + SASA_TLR_ – SASA_complex_) (Zhang et al., 2022). The simulated vaccine-TLR4 complex showed higher BSA values around 2,666.09 ± 162.77 Å^2^ as compared to the investigated vaccine-TLR2 model (2,276.82 ± 237.73 Å^2^) (Figure 5D). Notably, the vaccine-TLR4 system illustrated increased BSA beyond the 50 ns time window while greater fluctuations were demonstrated at the initial frames. The latter dynamic behavior indicated conformational stability, as well as more surface protein areas, which were covered at the second half of the simulation run and till its end. On the contrary, the vaccine-TLR2 complex depicted lower BSAs across the second half of the simulation run conferring fewer areas being covered. Nevertheless, the vaccine-TLR2 BSAs depicted steadier tones following the 50 ns time frame as compared to its initial times. Further tracking of the vaccine’s thermodynamic behavior was done by exploring the time-evolution conformational changes of the vaccine/TLR complex following the molecular dynamics run.

Conformational analysis through aligning the starting and final complex structures illustrated differential orientations for the bound ligand at the TLR binding interface (Figure 5E). Observed visually and correlated to the above obtained RMSD and RMSF trajectories, limited conformational and orientation changes were observed for the TLR proteins. A slight conformational shift was depicted for the TLR protomers showing minimal movement in its flexible loops (aligned RMSD 1.56 Å and 2.22 Å for TLR4 and TLR2, respectively). On the contrary, more dramatic conformational and orientational shifts were assigned to the vaccine protein with higher RMSD between its initial and final frame (5.33 Å and 11.57 in bound to TLR4 and TLR2, respectively). The latter RMSD values showed greater conformational/orientational shifts for the vaccine at the TLR2 interface. Significant drift (∼40 Å) was depicted for the vaccine’s carboxy-terminal and vicinal residue range (Ala327-to-Pro349) as well as the solvent-exposed amino acids (Thr100-Gly162) near the *N*-terminus. In the TLR4-bound complex, the latter vaccine’s high-flexible regions adopted more compacted conformations as they became more directed toward the lateral interface of the TLR4 protein at the end of the simulation time. On the contrary, the vaccine’s flexible regions at the TLR2 complex could not manage to adopt similar compactness the thing that was translated into higher distortions for the vaccine’s *C*-terminal side. Such dynamic behavior could explain the higher RMSF-related fluctuation patterns associated with these solvent-exposed residue regions as well as the higher RMSD/Rg tones for TLR2-associated vaccine over that at the TLR4 interface. The rest of the vaccine ternary structures at both TLR complexes showed limited conformational changes, particularly those being anchored deep at the TLR binding interface (inner concave surface).

**FIGURE 6:**
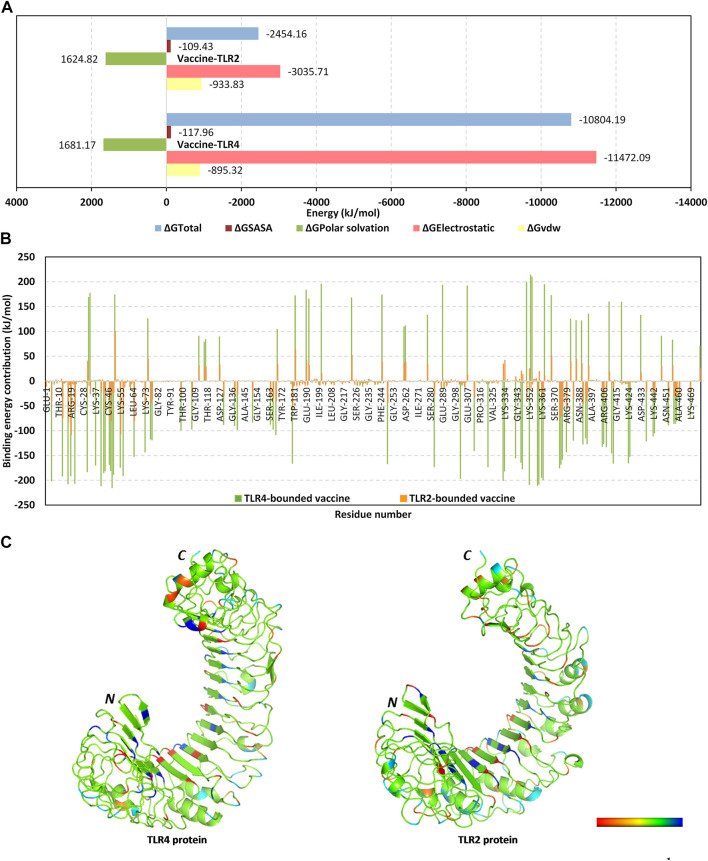
Binding interaction analysis for the simulated vaccine-TLR complexes across the molecular dynamics simulations. **(A)** MM_PBSA total free binding calculation and its constituting energy terms; **(B)** Per-residue MM_PBSA free binding energy contributions for multiepitope vaccine in terms of residues sequence numbering. Residue range for vaccine (*N*-terminal Glu1-to-Ser476 *C*-terminal), TLR4 (*N*-terminal Glu27-to-Cys627 *C*-terminal), and TLR2 (*N*-terminal Ser27-to-Pro575 *C*-terminal); **(C)** 3D-representation (Cartoon) for TLR regions corresponding to favored protein-protein affinities. Bold letters N and C; denote protein’s amino and carboxy terminals, respectively. Regions of TLR proteins are in spectrum colors from dark red for positive-valued ΔG kJ/mol (high-repulsive unfavored binding forces) up to dark blue for negative-valued ΔG kJ/mol (high-attractive favored binding forces).

Moving towards the vaccine-TLR binding affinity across the simulated thermodynamic trajectories, the MM_PBSA calculations revealed great concordance with the preliminary HawkDock-MM/GBSA docking binding scores. The trajectory-based MM_PBSA revealed higher negative average free binding interaction energies for the vaccine towards the TLR4 receptor interface as compared to those at the TLR2 side (ΔG_Total_ = −10804.19 ± 138.19 kJ/mol versus −2,454.162 ± 410.95 kJ/mol) (Figure 6A). At both vaccine-TLR complexes, pronounced dominance of the electrostatic potentials (ΔG_Electrostatic_) was depicted over the van der Waal interactions (ΔG_vdw_). The electrostatic potential energy contributions were more than 3-folds and 12-fold that of the van der Waal interactions at TLR4 and TLR2 interfaces, respectively. Notably, the polar solvation penalties (ΔG_Polar solvation_) at both TLR2 and TLR4 systems were almost comparable and were translated as repulsive forces against vaccine binding as well as compromised complex stabilities since binding is a solvent-displacement process (Barillari et al., 2007). Nevertheless, the extremely higher attractive electrostatic potentials as well as the total non-polar interactions (summation of ΔG_SASA_ and ΔG_vdw_) for the TLR4-associated complex over the TLR2 would have managed to overcompensate these unfavored repulsive polar solvation forces. All these furnished binding energy terms were translated into better vaccine affinity towards TLR4 over the TLR2 side. Finally, the relatively high total non-polar potentials could be correlated to the large hydrophobic receptor interface available for the multitope vaccine binding.

Exploring the residue-wise free binding energy contributions for the simulated complex were highlighted in Figures 6B, C. Higher negative-value energy contributions were assigned for the vaccine *N*-terminal residues as well as those constituting the extensive anti-parallel sheets. Repulsive positive-value energy contributions were limited to the residue area being exposed towards the solvent side, particularly *C*-terminal residues and vicinal amino acid region. Findings were consistent with the above-depicted RMSF values showing residues with high positive-value energy contributions to be correlated with high respective RMSF trajectories across the simulation time. Concerning the protein receptor, higher negative-value residue-wise energy contributions and lower repulsive positive contributions were assigned for TLR4 over the TLR2 unit. On the other hand, preferentially higher negative-value energy contributions were assigned for the *N*-terminal half of each TLR structure.

### The predicted immune response upon the potential vaccine injection

**FIGURE 7:**
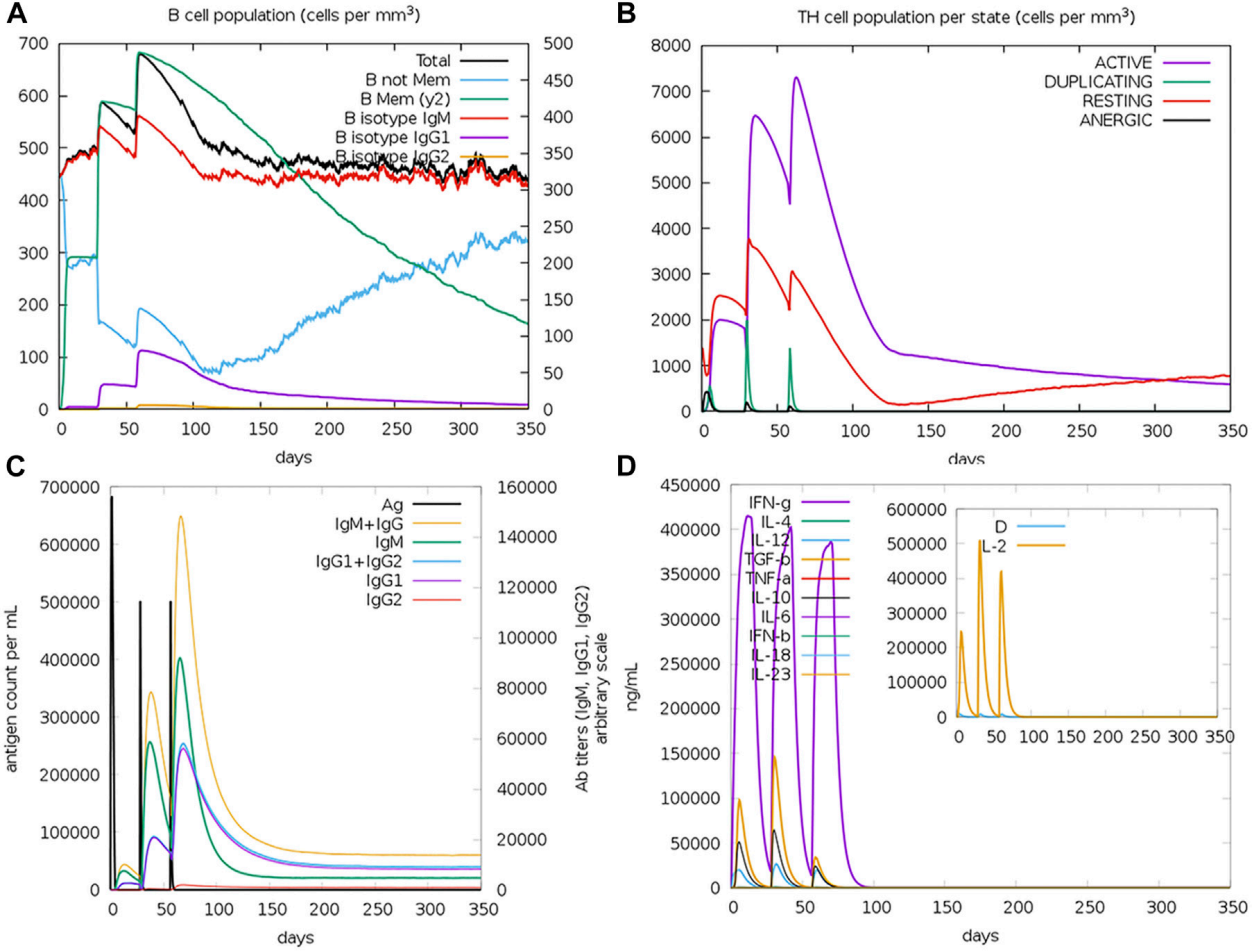
Assessment of the stimulated immune response upon the potential vaccine administration. **(A,B)** Show the population of B and T cells, respectively, while **(C,D)** demonstrate antibody and cytokine count in a response to vaccine administration.

The computational assessment through the C-ImmSim server predicted an overall satisfactory response with the successive doses of the potential multitope vaccine injection (Figure 7). It is clear that a high level of IgM + IgG antibodies was induced upon the multitope vaccine injection. Moreover, as we considered the induction of cytokines during T cell epitope filtration, it is not a surprise that the chimeric construct was predicted to stimulate several cytokines with INF-γ coming at the top of the stimulated cytokines list. Finally, the server output revealed that there was an obvious increase in both B and Th cell levels upon the potential vaccine injection where the highest level was observed after the second booster dose.

### Codon optimization for the potential vaccine construct

The output of the JCAT servers showed that the GC content of the improved sequence was 51.6% (the accepted range is between 30% and 70%. In addition to that, the calculated CAI value was 0.95, a value that indicates the suitability of the improved sequence to be easily expressed in *E. coli* k-12 (the CAI value ranges between 0 and 1 and the accepted range is between 0.8 and 1).

## Discussion

During the last few decades, the number of reports that study and analyze the phenomenon of antibiotic resistance showed a drastic increase with the continuous appearance of resistant strains of various pathogens to different antimicrobial agents (Birger et al., 2015; Zhuang et al., 2021; Bazaid et al., 2022). A more difficult situation occurs when the infection happens as a “coinfection” where more than one pathogen attacks the same host in a case that usually shows more complicated and life-threatening pathogenesis (Pasman, 2012). In the current study, we directed our interest to the coinfection status of *K. pneumoniae* and *P. aeruginosa* that were reported in severe pneumonia and chronic wound infection cases. As mentioned, with the continuous development of bacterial resistance mechanisms against the currently available antimicrobial agents, it became essential and a public health priority to find new solutions to this life-threatening condition. The continuous development in the next generation sequencing methodologies and the availability of public databases with huge genomic and proteomic data have directed our efforts to create novel techniques such as reverse vaccinology and immunoinformatics for the development of new vaccine candidates against life-threatening pathogens in an economic and time-saving manner (Kardani et al., 2020; Chakraborty et al., 2021). The scope of this newly designed vaccine has extended to include bacteria such as *Staphylococcus aureus* (Hajighahramani et al., 2017) and *Acinetobacter baumannii* (Touhidinia et al., 2021), viruses such as Lassa (Sayed et al., 2020) and Ebola (Kadam et al., 2020), and fungi such as *Candida auris* (Akhtar et al., 2021). Several studies moved forward and validated their epitope-based vaccines through wet lab experiments. Assessment of a multitope vaccine against *Echinococcus granulosus* revealed the presence of a significant difference in the weight of hydatid cysts between the Immunized group and the non-immunized one (Yu et al., 2021). Moreover, a designed epitope-based vaccine against uropathogenic *Escherichia coli* elevated the levels of IgG and IgA antibodies in the serum of immunized mice and offered high potency in the protection of the mice’s urinary tract (Hasanzadeh et al., 2020). A third study demonstrated the *in vivo* activity of a subunit vaccine against *A. baumannii* where the immunized mice experienced a decreased mortality rate n comparison to the control counterparts (Abdollahi et al., 2021).

Bacterial outer membrane proteins play important roles that support bacterial life and pathogenesis in the infected host. They are considered the first molecules to come into contact with the hosts’ cells, a characteristic that put these proteins an important candidate for vaccine development (Anand and Chaudhuri, 2016). These proteins have been selected as targets for vaccine design against several microorganisms including the current study targeted bacteria (Farhadi et al., 2015; Jahangiri et al., 2017; Dey et al., 2022). In addition to that, bacterial iron uptake proteins have been also selected in several trials as promising candidates for vaccine development against *K. pneumoniea* and *P. aeruginosa* (Nemati Zargaran et al., 2021; Hamad et al., 2023). Iron is an essential micronutrient for most bacteria that is used for many essential cellular processes where it is obtained from iron-chelating siderophores or directly from iron-containing host proteins. For Gram-negative bacteria, classical iron transport systems are composed of three major components; an outer membrane receptor, a periplasmic binding protein, and an inner membrane ABC transporter (Mosbahi et al., 2018). The current study aimed firstly to select promising protein candidates for vaccine development and for the above-mentioned points, we detected the protein targets in two main categories; outer membrane proteins and iron uptake one. The primary protein list was filtered, based on the antigenicity score, to select one protein per each class for our interested bacteria (*K. pneumoniae* and *P. aeruginosa*). Regarding *K. pneumoniae*, FepA and OmpF were selected as our final targets for epitope mapping. FepA is an integral outer membrane protein that is composed of 742 amino acid residues and transports ferric enterobactin where several reports revealed that immune responses against FepA can inhibit *K. pneumoniae* infections (Baghal et al., 2010; Lundberg et al., 2013). Baghal et al. generated a recombinant form of *E. coli* FepA and assessed for its immunogenicity in BALB/c mice and rabbits. The results demonstrated that FepA stimulated a significant response that protected the tested animals against *K. pneumoniea*. Our second protein target for *K. pneumoniae* was OmpF, a protein that plays roles in both antimicrobial resistance and bacterial virulence (Tsai et al., 2011). Moving to *P. aeruginosa*, HasR and OprF were our final protein candidates where the former was reported for its essential roles in sensing and transport of the extracellular heme (Dent and Wilks, 2020), while the later is an essential enzyme for the bacterial full virulence (Fito-Boncompte et al., 2011) and had a role in the resistance to macrophage clearance during acute infection (Moussouni et al., 2021).

The approach of computational vaccine design has been employed in several previous trials where some reports were only interested in the nomination of potential vaccine candidates (Nagpal et al., 2018; Mehmood et al., 2020) or started with a predefined virulent protein as potential vaccine candidates (Elhag et al., 2020; Mahapatra et al., 2021) and others extended their scope to include the epitope mapping and the construction of a multitope construct after protein candidates nomination (Cuscino et al., 2022; Wang et al., 2022). On the other hand, the current study exploited the option of mono epitopes integration and fused the mapped epitopes after the filtration of several vaccine candidates of two bacteria, *K. pneumoniae* and *P. aeruginosa*, to create a multitope construct with potential activity against the increasingly reported coinfection cases with those resistant bacteria. Moreover, the current study not only extended the scope of targeted bacteria but also provided a computational validation for the docking study through a molecular dynamics analysis, whereas other studies targeted one microorganism and stopped at the epitope mapping stage (Zargaran et al., 2021). The current study has employed several tools to design and computationally assess the potential vaccine construct. Regarding the process of epitope mapping, we employed the NetMHCpan EL 4.1 prediction method for T cell epitopes prediction, a tool that exploits tailored machine learning strategies to integrate different training data types, resulting in outperforming other prediction methods (Reynisson et al., 2021). For B cell epitopes assessment, we employed BepiPred-2.0 as a prediction method as it outperformed several available tools when they were assessed on a large collection of linear epitopes downloaded from the IEDB database (Jespersen et al., 2017). The current study utilized the Robetta server to predict the 3D structure of the established multitope construct, a server that is continually evaluated through CAMEO (Continuous Automated Model EvaluatOn) therefore Robetta has been and continues to be among the most consistent top-performing servers and consequently, it has been employed in the 3D structure prediction of multitope constructs in several similar studies (Khan et al., 2021; Sethi et al., 2022; Suleman et al., 2022; Bakkari, 2023).

After the filtration steps of the protein candidates, the selected ones were mapped for their B and T cell epitopes. Reliance on the epitopes for the vaccine design, instead of the whole protein, provides an important advantage of targeting only the antigenic parts of the protein and lessening the probability of allergic reactions (Oyarzun et al., 2021). On the other hand, the single epitopes may suffer from limited immunogenicity and for this purpose, we planned to integrate the single epitopes from each of the filtered proteins to initiate a multitope vaccine construct that would have an improved antigenicity in comparison to the single epitopes (Parvizpour et al., 2020). It is worth mentioning that the usage of suitable amino acid linkers to initiate the multitope potential vaccine construct is an important step as these linkers assure the appropriate separation of the integrated single epitopes *in vivo* (Hajighahramani et al., 2017). Starting with EAAAK, it was used to improve the bi-functional catalytic activity and enhance the fusion protein stability. Moreover, GPGPG, was selected for its ability to induce HTL immune response and the ability to break the junctional immunogenicity, resulting in individual epitopes’ restoration of immunogenicity. The final linker, KK, was employed because of its ability to bring the pH value close to the physiological range (Sami et al., 2021; Soltan et al., 2022b). Additionally, PADRE peptide and β-defensin were incorporated in the final multitope construct to enhance CD4^+^ T cell responses and potentiate the immune response, respectively (Mei et al., 2012; Ghaffari-Nazari et al., 2015). Following the integration for the potential multitope vaccine construction, it was analyzed for its characteristics and was found to be soluble upon over-expression, antigenic, non-allergen, non-toxic, and stable (with an instability index less than 40). Collectively, the output of this stage assessment moved our study to the next steps of tertiary structure prediction and docking analysis.

Accumulated evidence correlated the important role of both TLR2 and TLR4 immune receptors within the *K. pneumoniae*-directed host defenses (Paczosa and Mecsas, 2016). Both reducted mortalities and *K. pneumoniae*-host disseminations were associated with TLR4 and TLR2 immune responses within the pneumonia mouse model (Wieland et al., 2011). While TLR4 would impair and control the infection spreading throughout the initial stage of infection, the conger immune receptor, TLR2, was reported beneficial for reducing inflammatory levels associated with the infection. Subsequently, TLR2 was solely reported to control and prevent bacterial expansions within the later stages of infection. In these regards, several reported studies tend to computationally investigate the affinity of both immune receptors in a way to predict their promising role (Jeon et al., 2017; Dar et al., 2019; Allemailem, 2021). In our study, molecular docking-coupled dynamic simulations demonstrated significant binding of constructed multi-epitope vaccine towards the TLR2 and to a higher extent to the TLR4 interface. Vaccine binding with either TLR4 or TLR2 receptor was residue-wise dependent since differential stability, fluctuation patterns, and binding energy contributions were assigned for each protein down to its amino acid level. Generally, TLR exists at the stage before downstream signal transduction in the C-shaped horse-shoe architecture (Park et al., 2009; Ohto et al., 2012). The inner concave surface of TLRs has been successfully investigated within current literature as the stable interface for different multi-epitope vaccines targeting different types of microbial organisms (Soltan et al., 2021; 2022a; Cuscino et al., 2022). Focusing on vaccines targeting *K. Pneumoniae*, Dar et al. reported successful affinity for their immunoinformatics-designed multiepitope vaccine towards the inner concave interfaces of both TLR4 and TLR2 (Dar et al., 2019). Notably, our CPORT-based prediction for TLR binding interfaces came in agreement with Dar et al. study suggesting several hot-spot residues at the TLR interface for directing vaccine binding. Moreover, the furnished CPORT-based analysis predicted the favored binding of the vaccine *via* its highly packed anti-parallel β-sheets ternary structure the thing that was adopted throughout the relevant selection of the docked binding mode.

The preferential binding of multitope vaccine to the TLR target was demonstrated through a multi-level stability analysis. The RMSD analysis was significant for showing limited conformational changes and superior relative stability depicting steady tones across the simulation times. On general bases, RMSD trajectories provide accurate measurement regarding a molecular deviation from its reference structure at the beginning of the molecular dynamics simulations (Arnittali et al., 2019). High protein RMSDs usually correlate to significant conformation alterations and instability, while as for ligands they confer compromised ligand-target affinity and ligand-pocket accommodation (Liu et al., 2017). Tone from ROG came in good translation for the RMSD findings, since these parameters depicted inherited stability, compactness as well as tight contact distances for simulated proteins. Generally, lower ROG values with limited fluctuations suggested optimum structural compactness in terms of favored inter- or intra-molecular interactions (Likić et al., 2005). The vaccine’s higher fluctuating RMSDs, ROGs, and RMSFs than the corresponding TLR could be reasoned for the differential protein ternary structures. The incorporation of long *α*-helices with flexible *β*-loop connections within the vaccine’s designed structures could reason for higher inherited flexibility throughout the simulation runs. Additionally, the initial docking pose with an extended *C*-terminal would favor a great conformational shift for final convergence into more stable compacted conformations. On the contrary, the densely packed TLR orchestra with shoe-like structure would advent from its several highly ordered parallel *β*-sheets the thing that would be correlated with limited flexibility and thermodynamic fluctuations. Comparable flexibility patterns were also depicted within several reported studies investigating the potential binding affinity of peptide-based vaccines toward microbial TLRs (Chauhan et al., 2019; Sanches et al., 2021). Comparative binding of our constructed vaccine was highlighted preferential for TLR4 over TLR2 interface. This was illustrated through lower values and minimal fluctuations for RMSDs, ROGs, RMSFs, and BSA tones regarding the binding proteins within the TLR4 complex system over TLR2 one. Our depicted TLR4-directed favored binding was in good agreement with reported data in the study by Cuscino et al. evaluating a bioinformatic-designed multiepitope vaccine targeting carbapenemase-releasing *K. pneumoniae* strains (Cuscino et al., 2022).

Stability patterns were successfully translated into high negative free binding energy. Both MM_GBSA and MM_PBSA binding energy calculations for respective docked and molecular dynamics complexes illustrated the predominance of electrostatic potentials and polar residue energy contributions for the vaccine towards the TLR site. Greater electrostatic negative values, suggesting stronger binding affinities, were consistent with reported results of other research groups investigating vaccines of other microorganism origins towards different TLRs, including our investigated ones TLR4 and TLR2 (Dar et al., 2019; Soltan et al., 2022a). It is worth noting that, the here simulated vaccine was in dynamic motion at the TLRs interface the thing which is consistent with the reported thermodynamic behavior of various protein-protein complexes (Zhang et al., 2016; Zhang and Buck, 2017; Sami et al., 2021). Notably, the more dynamic behavior of the vaccine as compared to TLRs would suggest furnishing less unfavored entropy on binding than those obtained with complexes where both or either one partner is significantly rigid (Peccati and Jiménez-Osés, 2021). Additionally, thermodynamic flexibility could also be of extra advent since higher conformational changes could also be seen with a vaccine for accommodating more compact conformation at TLR lateral interface. Additionally, depicted vaccine dynamic behaviors were likely accompanied by indirect hydrogen bonding with water molecules at or even near the interface bridging such as polar interactions the thing that would overcompensate the polar solvation entropic penalties as a result of displacing highly ordered water molecules at interacting protein surfaces. The latter was seen with several protein-protein complexes where one partner is of more solvent exposure (Soltan et al., 2021; 2022b).

## Conclusion

The continuous increase in the rates of bacterial antibiotic resistance makes it a public health concern to develop novel solutions. Reliance on the computational tools to design and *in silico* evaluate potential vaccine candidates is a promising technique that witnessed great development in the last few years. With the advantages of being a time and cost-saving approach, we selected 4 protein candidates from the proteome of *K. pneumoniae* and *P. aeruginosa* and mapped B and T cell epitopes for these candidates. The most promising mono epitopes were integrated into a multitope construct which in turn was evaluated for its physicochemical, immunological, and binding characteristics with 2 TLRs. Results of the computational assessments nominated our multitope construct as a potential vaccine against the coinfection status of *K. pneumoniae* and *P. aeruginosa*. Future wet lab validation is an essential next step to validate the current findings.

## Data Availability

The original contributions presented in the study are included in the article/Supplementary Material, further inquiries can be directed to the corresponding author.
