# Supplementary material for: Integration of immunoinformatics and cheminformatics to design and evaluate a multitope vaccine against Klebsiella pneumoniae and Pseudomonas aeruginosa coinfection
Source: Front Mol Biosci. 2023 Feb 14;10:1123411. doi: 10.3389/fmolb.2023.1123411 (PMC9999731; doi:10.3389/fmolb.2023.1123411)
Supplement: Supplementary file 1 [file Table1.DOCX]

**Integration of Immunoinformatics and Cheminformatics to Design and Evaluate a Multitope Vaccine against *Klebsiella pneumoniae* and *Pseudomonas aeruginosa* Coinfection**

**Supplementary figures**

**
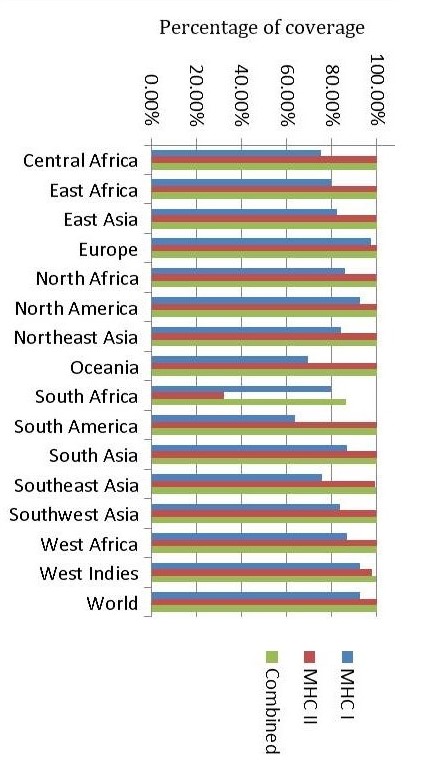
**

Supplementary figure 1: Population coverage with MHCI, MHCII, and combined MHC epitopes based on their respective HLA binding alleles


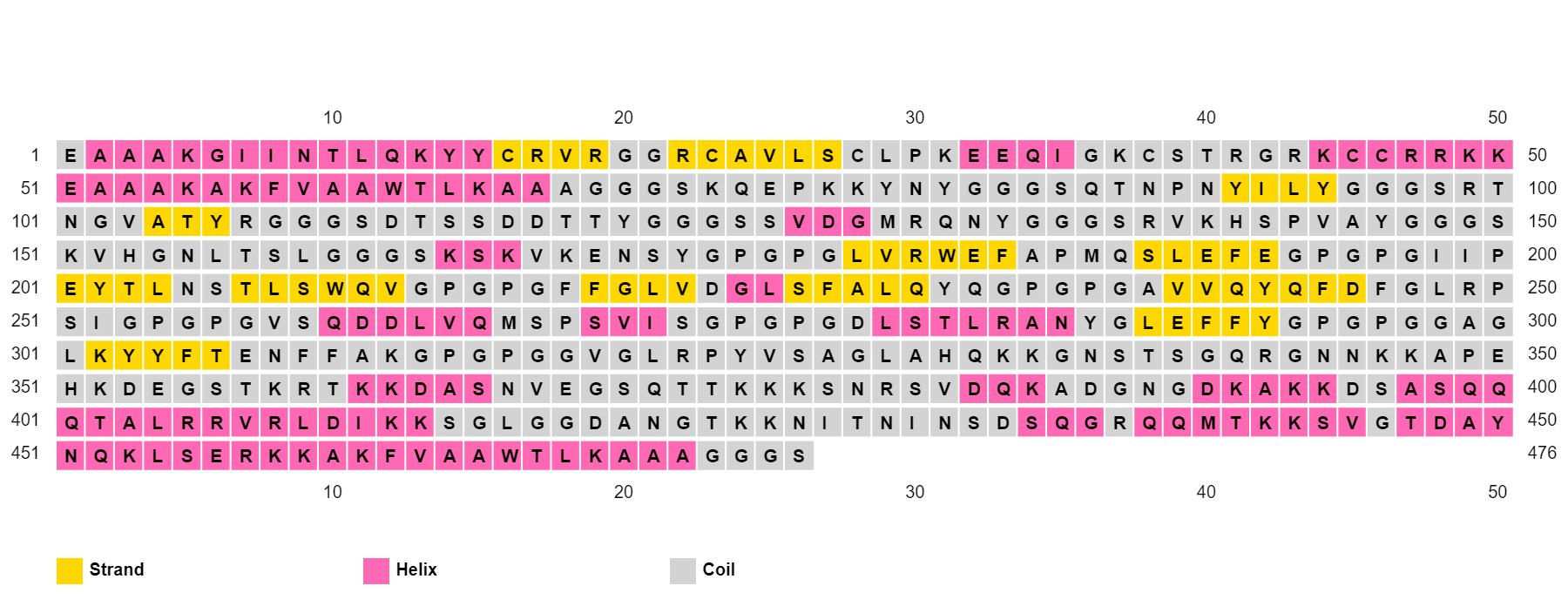


Supplementary figure 2. Secondary structure prediction of designed multitope vaccine using PESIPRED server.


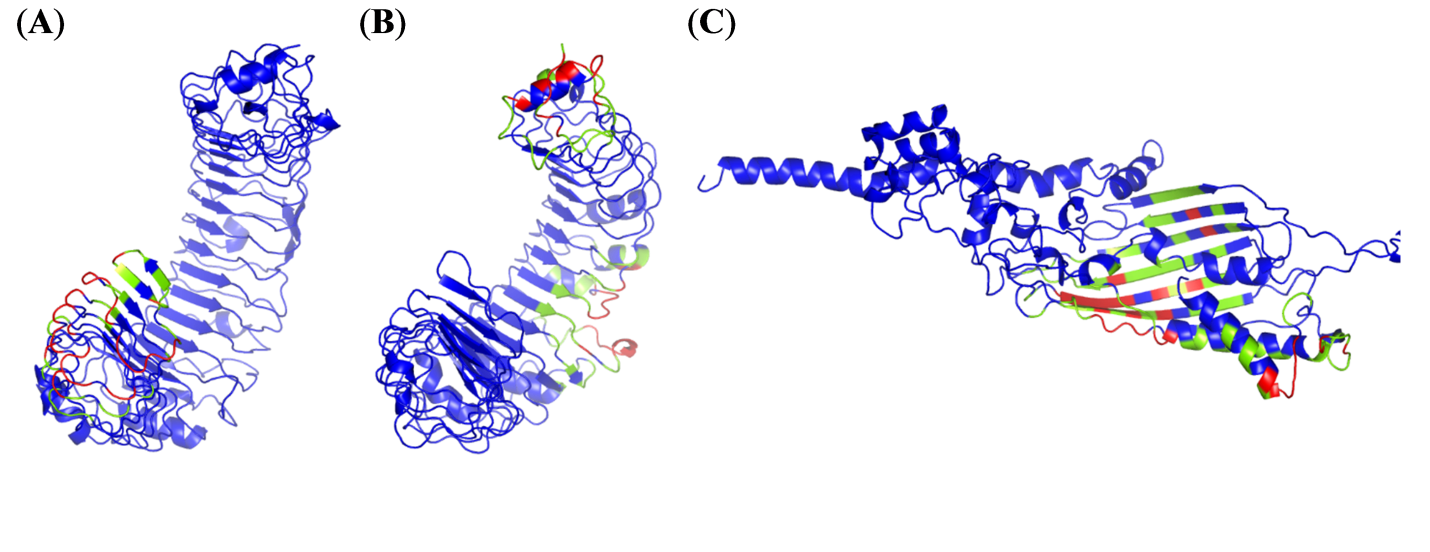


Supplementary figure 3. Predicted active and surrounding residues for investigated proteisns. (A) TLR4, (B) TLR2, (C) our constructed multiepitope vaccine. Proteins are shown in cartoon representation with red and green colored regions being correlated to the active and surrounding residues, respectively, on B-factor (temperature) column.

**Supplementary tables**

Supplementary table 1. Physicochemical characteristics of the designed multitope vaccine.

| Physicochemical characteristic | Molecular weight | Theoretical  pI | Extinction  coefficient | Instability  index |  | GRAVY | Aliphatic  index |
| --- | --- | --- | --- | --- | --- | --- | --- |
| Score | 49.83 kDa | 9.86 | 52175 M^-1^ cm^-1^ | 33.24 |  | -0.742 | 53.53 |
